# HLAII peptide presentation of infliximab increases when complexed with TNF

**DOI:** 10.3389/fimmu.2022.932252

**Published:** 2022-09-13

**Authors:** Andrea Casasola-LaMacchia, Robert Joseph Seward, Sophie Tourdot, Matthew Willetts, Gary Kruppa, Michael J. Agostino, Gabrielle Bergeron, Nathalie Ahyi-Amendah, Andrew Ciarla, Zhaojiang Lu, Hai-Young Kim, Timothy P. Hickling, Hendrik Neubert

**Affiliations:** ^1^ BioMedicine Design, Worldwide Research, Development and Medical, Pfizer Inc., Andover, MA, United States; ^2^ Bruker Daltonics, Billerica, MA, United States; ^3^ Pfizer Digital, Pfizer Inc., Andover, MA, United States; ^4^ Analytical Research and Development, Biotherapeutics Pharmaceutical Sciences, Pfizer Inc., Andover, MA, United States

**Keywords:** CD4+ T-cells, HLAII, dendritic cells, TNF antagonist, presented peptides, immunogenicity, timsTOF mass spectrometry, immunopeptidomics

## Abstract

CD4+ T-cell activation through recognition of Human Leukocyte Antigen II (HLAII)-presented peptides is a key step in the development of unwanted immune response against biotherapeutics, such as the generation of anti-drug antibodies (ADA). Therefore, the identification of HLAII-presented peptides derived from biotherapeutics is a crucial part of immunogenicity risk assessment and mitigation strategies during drug development. To date, numerous CD4+ T-cell epitopes have been identified by HLAII immunopeptidomics in antibody-based biotherapeutics using either their native or aggregated form. Antibody-target immune complexes have been detected in patients with ADA and are thought to play a role in ADA development by enhancing the presentation of CD4+ T-cell epitopes at the surface of antigen presenting cells (APCs). The aim of this study was to investigate the effect of biotherapeutic antibody-target immune complexes on the HLAII peptide presentation of biotherapeutics in human primary monocyte-derived dendritic cells (DCs). The trimeric tumor necrosis factor (TNF) and its biotherapeutic antagonists infliximab (INFL), adalimumab (ADAL), and a single armed Fab’ were used as a model system. The HLAII immunopeptidome of DCs loaded with antagonists or their immune complexes with TNF was analyzed by trapped ion mobility time-of-flight mass spectrometry (timsTOF MS) leading to the identification of ~ 12,000 unique HLAII-associated peptides per preparation. Anti-TNF sequences were detected at a median of 0.3% of the total immunopeptidome, against a majority background of peptides from endogenous and media-derived proteins. TNF antagonist presentation spanned the variable and constant regions in a widespread manner in both light and heavy chains, consistent with previously discovered HLAII peptides. This investigation extends the collection of observed HLAII peptides from anti-TNF biotherapeutics to include sequences that at least partially span the complementary determining regions (CDRs), such as the LCDR1 for both INFL and ADAL. Although antagonist presentation varied significantly across donors, peptides from both bivalent antagonists INFL and ADAL were more highly presented relative to the Fab’. While TNF immune complexes did not alter overall HLAII presentation, a moderate increase in presentation of a subset of peptide clusters was observed in the case of INFL-TNF, which included HCDR2, HCDR3 and LCDR2 sequences.

## Introduction

Unprecedented progress has been made with the development of an increasing diversity of protein-based biotherapeutics leading to new treatments for human diseases such as cancers, autoimmune and inflammatory disorders (1–5). Monoclonal antibodies (mAbs) and their derivatives constitute a major group of biotherapeutics characterized by their high specificity of targeting. Advances in recombinant DNA technologies enable their discovery and upscaled production (4–6).

However, a major challenge to the development of mAbs is the unwanted anti-drug immune responses, i.e. immunogenicity that, if not identified early, can result in termination of late-stage clinical programs, which is both costly, inefficient and incurs delays for patients who need novel treatment options (6–8). Indeed, development of ADA may trigger hypersensitivity reactions and decrease efficacy (9, 10), by accelerating clearance of the drug in circulation upon formation of immune complexes or by blocking or neutralizing the target site binding (11–30).

The sequences from the protein therapeutic that are presented by the HLAII of APCs can constitute a liability from an immunogenicity perspective if they trigger CD4+ T-cell activation leading to the generation of ADA. Thus, the identification and removal of such sequences is an important *in vitro* analytical strategy that complements T-cell epitope discovery and deimmunization strategies for immunogenicity risk assessment and mitigation (31, 32). To this end, the *in vitro* characterization of the immunogenicity sequence liabilities of TNF antagonists, which are some of the most widely used biotherapeutics, has been reported, including the use of HLAII immunopeptidomics, also known as MHC-associated peptidome proteomics (MAPPS) (32–37). For example, the chimeric infliximab (INFL), which has been shown to have high unwanted immunogenicity rates in the clinic, exhibits HLAII presentation of peptide clusters including those from the variable regions (33, 34, 37, 38). Consistently, *in vitro* activating CD4+ T-cell epitopes with these INFL peptides largely overlap with high-affinity binders to HLA-DR, the most abundant HLAII (33, 39). That is also the case of the major T-cell activation epitopes from ADAL spanning the HCDR2, which were previously found to be presented by the HLAII using immunopeptidomics (32). INFL and ADAL aggregated by physical stress, including heat, have been demonstrated to influence DC activation, including proinflammatory cytokine signaling and, in certain cases, HLAII expression leading to increased internalization of antibody and peptide presentation (40–43).

The characterization of the effect of complexes formed between the anti-TNF biotherapeutics and TNF, on HLAII peptide presentation has not yet been studied. However, this information is expected to further contribute to the understanding of the immunogenicity of these molecules. Previously, anti-TNF immune complexes with TNF have been detected in patients with rheumatoid disorders under treatment with INFL and ADAL (44, 45). *In vivo* models have suggested that these INFL-TNF immune complexes indeed contribute to mounting an ADA response and production of inflammatory cytokines, an effect also observed with ADAL-TNF immune complexes in macrophages (44, 46).

The work presented herein sought to extend these investigations by using mass spectrometry to detect differences in HLAII presentation of anti-TNF therapeutic-derived peptides in the context of complex formation. The characterization of HLAII immunopeptidomes from DCs loaded with TNF antagonists alone or as immune complexes with TNF enabled the comparison of peptides presented from INFL, ADAL and a single arm Fab’. To this end, *in vitro* complex formation of antagonists with TNF was confirmed by dynamic light scattering (DLS), then DCs were loaded, cultured and harvested prior to HLAII immunopurification, acid elution and timsTOF MS. HLAII peptides derived from the three antagonists INFL, ADAL and Fab’ were detected and analyzed for differences between complex and non-complex conditions. Predominant HLAII presentation of INFL and ADAL-derived peptides was detected. In contrast, Fab’-presentation was found limited to a small percentage of donor-sets. Furthermore, DCs treated with *in vitro* formed immune complexes increased INFL-presentation, while presentation of peptides from ADAL did not change overall as analyzed by qualitative and quantitative assessments.

## Experimental procedures

### Experimental design and statistical rationale

**Figure 1:**
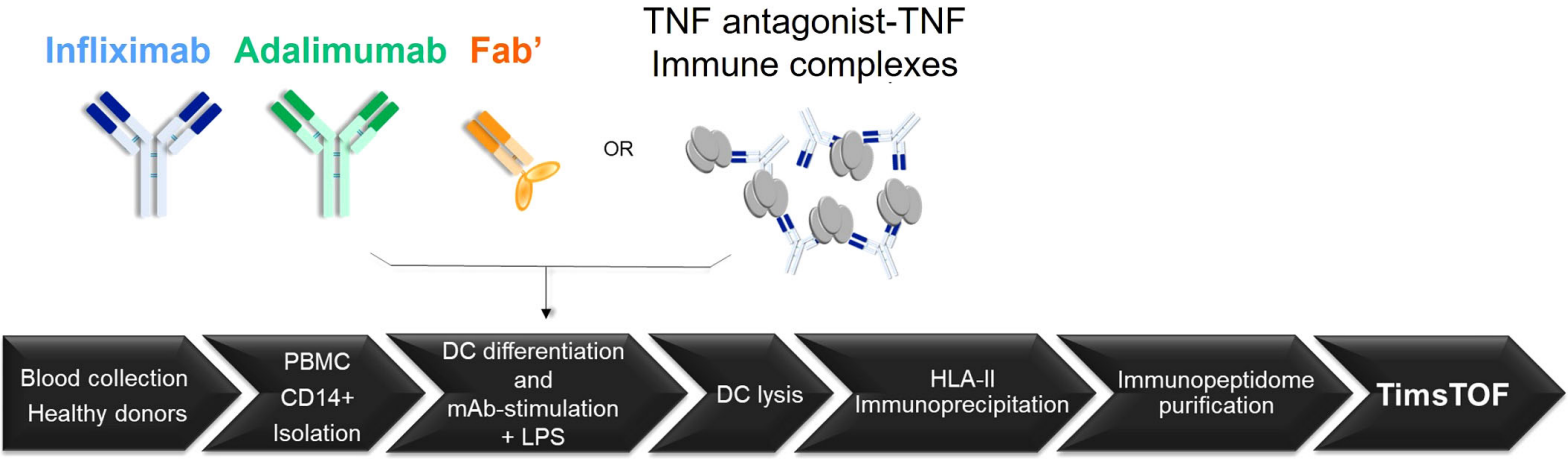
HLAII immunopeptidomics workflow for the identification of peptides presented from TNF antagonists by DCs. Freshly isolated PBMCs collected from fourteen healthy donors were isolated by density separation for subsequent CD14+ monocyte-purification. CD14+ monocytes were cultured and differentiated into DCs for six days. DCs were loaded with TNF antagonists or their preformed TNF immune complexes at day 5, followed by LPS stimulation for 22 h. DCs were harvested, washed and lysed to obtain whole protein extracts for HLAII immunoprecipitation using a pan-HLAII antibody. The HLAII immunopeptidome was purified by filtration and by C18 prior to LC-MS/MS analysis using trapped ion mobility time-of-flight mass spectrometry (timsTOF MS).

To investigate the influence of immune complexes of TNF with TNF antagonists on antigen presentation, the HLAII immunopeptidomes of monocyte derived DCs from fourteen individual subjects were analyzed (**Figure 1**). Per donor, nine DC preparations with different conditions leading to independently isolated HLAII immunopeptidomes were assessed corresponding to three major groups: a) controls: unstimulated (UNSTIM), LPS- and TNF-induced DCs; b) antagonists only: INFL, ADAL and Fab’ and c) TNF immune complexes: INFL-TNF, ADAL-TNF and Fab’-TNF. *In vitro* formation of TNF immune complexes with INFL, ADAL and Fab’ antagonists was analyzed and confirmed by diameter-size measurements using dynamic light scattering (DLS). TNF immune complexes were freshly prepared prior to incubation with DCs and analyzed in fourteen independent experiments, i.e., every time prior to administering them to DCs derived from each of the fourteen donors. A total of 126 immunopeptidomes were analyzed. The *MS/MS spectra*, peptide-spectrum matches (*PSM*s) upon database searching, and *unique peptide identifications* were compared between treatments (n=9) and across donors (n=14). T-test, Wilcoxon marched-pairs signed rank test, Mann-Whitney and two-way ANOVA were used in this study. Normalization based on the peak areas of identified endogenous peptides was carried out using *dataMAPPs*, an immunopeptidomics data processing and visualization platform which employs a ‘global rank-invariant set normalization’ (GRSN) procedure using signals of the most invariant peptides within datasets per donor (37).

### *In vitro* TNF immune complex formation and biophysical assessment by DLS

TNF (R&D Systems, 210-TA/CFMTO) was stored in 0.5 mL single use aliquots at -80°C and thawed on ice for 45 min before use. For immune complex formation, the TNF antagonists INFL (infliximab, Janssen, Leiden, Netherlands), ADAL (adalimumab, Abbvie, Chicago, IL, USA) and a polymer-conjugated Fab’ were prepared at 0.03 mM, independently mixed and incubated for 30 min at room temperature with 0.02 mM TNF in PBS at 1:1 volume to yield a total volume of 500 µL. As controls, TNF or each of the antagonists were incubated in parallel on their own under the same conditions, constituting the following test articles: INFL, ADAL, Fab’, INFL-TNF, ADAL-TNF and Fab’-TNF. Three replicates (9 µL each) were collected at 25°C by dynamic light scattering (DLS) using UNCLE (Unchained labs, Pleasanton, CA, USA). The hydrodynamic diameters were analyzed with UNCLE v4.10 software. The remaining TNF immune complexes were used immediately on primary monocytic DCs.

### Differentiation of primary monocyte-derived DCs, test article loading and LPS stimulation *in vitro*

DCs were generated as previously described (39). Briefly, CD14+ monocytes were isolated from peripheral blood mononuclear cells (PBMCs) collected with informed consent from fourteen healthy donors (D1237, D1095, D2052, DCE00, D1802, D1091, D1265, D1761, D2308, D2035, D1170, D1289, D1214; STEMCELL Technologies, Vancouver, BC, Canada). PBMCs from half-leukopaks were isolated by Ficoll density separation (GE Health Sciences) and CD14+ monocytes were isolated by magnetic bead-based cell separation (MACS, Miltenyi Biotec, MA, USA) using bead-conjugated anti-CD14 antibodies and magnetic cell separation columns according to the manufacturer’s protocols. Monocytes were cultured at a density of 5×10^5^ cells/mL in RPMI medium supplemented with 10% (v/v) FBS, GM-CSF (100 ng/mL) and IL-4 (17 ng/mL) for 6 days. Test articles were added at 50 µg/mL to the monocytic DCs in culture on Day 5, followed by LPS stimulation (32 ng/mL) after 4 h, resulting in nine DC cultures for each donor (UNSTIM, LPS, TNF, INFL, ADAL, Fab’, INFL-TNF, ADAL-TNF, Fab’-TNF). DCs were harvested 22 h post-LPS addition, washed three times with PBS, counted, and stored at –80°C until further processing.

### HLAII immunopeptidomics

Whole protein lysates from DCs were prepared as described (39). Briefly, pelleted cells were partially thawed on ice and resuspended in 1 mL cold detergent containing lysis buffer including protease inhibitors (20 mM Tris, 150 mM NaCl, 1 mM PMSF; Sigma, A3428-10MG, 5 μg/mL Aprotinin; Sigma, A3428-10MG, 10 μg/mL Pepstatin A; Calbiochem, 516481-5MG, 10 μg/mL Leupeptin; Sigma, L5793-5MG, 1% w/v CHAPS). Lysates were incubated for 1 h with end-to-end rotation at 4°C, pelleted at 2,500 g for 5 min and the supernatant was recovered. Immunoprecipitation of total HLAII was carried out by adding 400 μg of anti-panHLAII antibody (purified from anti-HLA-DP/DQ/DR antibody ATCC IVA12 HB-145 hybridoma). Lysates were incubated with antibody overnight at 4°C with end-to-end rotation. 200 μL of 50% Protein G agarose bead slurry (Thermo Fisher Scientific, 22852) were added and incubated with end-to-end rotation at 4°C for 2 h. Bead bound complexes (HLAII-mAb-beads) were collected on 96-well filter plates (Agilent, 204495-100) and flow-through was collected in 2 mL well collection plates (Agilent, 201240-100). HLAII-mAb-beads in each well were washed twice with lysis buffer 0.5% v/v CHAPS followed by six washes with 20 mM Tris pH 8 without inhibitors in a positive pressure-96 processor (Waters, Milford, MA). HLAII peptide complexes were eluted twice from the beads with 150 µL of 10% acetic acid and HLAII-bound peptides were collected in a Lobind 96-well plate and further transferred to pre-conditioned 10,000 MWCO filters (Millipore, MRCPRT010) for membrane separation at 14,000 RCF for 50 min. Peptides were then processed in Sep-Pak tC18 pre-conditioned SPE 96-well plates (5 mg sorbent per well at 37-55 µm; Waters, 186002318). Eluates were loaded to the tC18 plates and washed twice with 500 µL of 0.1% TFA. A Lo-Bind 96-well collection plate was used to retrieve the peptide samples following incubation with 150 µL of 50% ACN, 0.1% TFA for 5 min before application of positive pressure. Elution was repeated and the second eluate was collected into the same plate (~300 µL total volume). The tC18 plate situated above the collection plate was centrifuged for 1 min at 2,500 RCF to recover any residual eluate. Each sample was then split into four 75 µL aliquots in 1.5 mL Eppendorf tubes. Individual aliquots were dried in a SpeedVac and stored at -80°C until use.

### Liquid chromatography–tandem mass spectrometry (LC-MS/MS)

A dried aliquot per sample was equilibrated on ice and resuspended in 7 µL 3% ACN, 0.1% FA. Peptides were chromatographically separated and analyzed using a nanoElute nano HPLC system coupled to a trapped ion mobility time-of-flight mass spectrometer (timsTOF Pro, Bruker Daltonics, Billerica, MA, USA). A 6.5 µL aliquot of each sample was loaded after equilibration for 3.6 min with mobile phase A (approximately 6 column volumes) onto a 25 cm, 75µm C18 Aurora column (IonOpticks, Australia) at a flow rate of 400 nL/min using mobile phase A (water plus 0.1% formic acid) and mobile phase B (acetonitrile plus 0.1% formic acid). Sample loading took 27 min. The chromatographic gradient was as follows: 2 to 25% B over 60 min, then 25 to 37% B over 7 min, followed by 67 - 80% B over 10 min, then maintain at 80% B for 10 min before returning 2% B. Total run time was 117.6 min.

The timsTOF Pro was operated in PASEF mode with the following settings: mass range 100 to 1,700 m/z, 1/K0 Start 0.6 V·s/cm2, End 1.6 V·s/cm2, ramp and accumulation were set to 166 ms, capillary voltage 1,600 V, dry gas 3 l/min, dry temp 200°C, PASEF settings: 10 MS/MS frames (1.89 seconds duty cycle), charge range 0-5, active exclusion for 0.4 min, target intensity 20,000, intensity threshold 2,500, CID collision energy was 20 to 59 eV depending on the inverse mobility of the precursor. A polygon filter was applied to the *m/z* and ion mobility pane to select features most likely representing peptide precursors rather than singly charged background ions.

### Database search, data normalization and visualization

MS/MS data were searched against the human proteome with added bovine proteins and light chain (LC) and heavy chain (HC) sequences from TNF antagonists. The Swiss-Prot protein databases from *Homo sapiens* and *Bos taurus* included 20,352 and 6,009 entries, respectively, and were downloaded from UniProt on 2020-01-31 (Supplementary Material_Databases) and analyzed by Byonic version 10.0 (Protein Metrics, California, USA) and PEAKS Studio X Pro (Bioinformatics Solutions, Toronto, CAN). TimsTOF data files were imported into both database search platforms with a precursor mass error tolerance of 30 ppm and the fragment mass error tolerance to 0.05 Da without enzyme specificity. Cysteinylation, oxidation (M), deamidation (NQ), pyro-glu (QE) were included as variable modifications. For PEAKS database searches, 1% FDR threshold was used (target-decoy approach). HLAII immunopeptidomes from the nine conditions were analyzed by Byonic. PEAKS data was used for additional evaluation and verification of test article derived peptides as well as normalization using the *dataMAPPs* pipeline (Supplementary tables_Final_PeptideIdentifications, MassIVE.ucsd.edu). Briefly, the PEAKS database search results were individually exported as DB psm.csv files and analyzed by a modified version of *dataMAPPs* [(37), R-code provided in Supplementary material). Detected TNF antagonist sequences across donors and conditions were visualized using heat maps (*heatMAPPs*) following two rounds of peak intensity normalization by the global rank-invariant set normalization (GRSN) procedure in *dataMAPPs*, which was applied with the default parameters listed in Analyze.R file (**Supplementary material**).

## Results

### Size characterization of TNF antagonists and *in vitro* formed complexes

**Figure 2:**
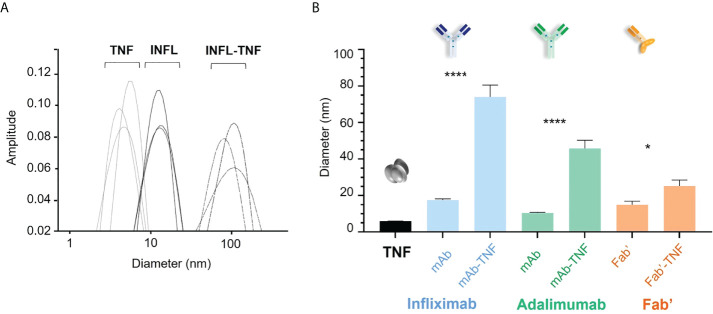
Size characterization of TNF antagonists and immune complexes of TNF with antagonists by dynamic light scattering (DLS). **(A)** Representative hydrodynamic diameter profile of TNF, INFL and INFL-TNF as measured by DLS. Each line denotes one of three technical replicates for each condition, which were then ran in fourteen independent experiments, i.e., one for each donor. **(B)** Size comparison of TNF (black), TNF antagonists and their complexes with TNF; INFL and INFL-TNF (blue), ADAL and ADAL-TNF (green) and Fab’ and Fab'-TNF (orange), where each bar correspond to the mean and the standard error of mean (SEM) from fourteen independent experiments (n=14, ****p < 0.0001, *p < 0.05).

The hydrodynamic diameter of TNF and each antagonist was compared to that of freshly formed complexes with TNF in fourteen independent experiments by DLS. Average diameter distribution for TNF was detected at 5.9 ± 0.4 nm (median 5.9 nm) and average sizes of antagonists corresponded to 17.5 ± 2.4 nm (median 16.9 nm), 10.5 ± 1.0 (median 10.4 nm), and 14.9 ± 7.2 nm (median 13.4 nm) for INFL, ADAL and Fab’, respectively (**Figure 2A**). TNF antagonists formed immune complexes of varying sizes when incubated with the trimeric cytokine at 3:2 molar ratio. The largest complexes in the analyzed panel were formed by INFL-TNF at 74.0 ± 24.6 nm (median 63.7 nm), which were followed by ADAL-TNF at 45.8 ± 16.8 nm (median 41.4 nm). In contrast, the single-armed Fab’-TNF only showed a moderate size increase between immune complexes and the control, corresponding to 25 ± 12 nm (median 22.7 nm) (**Figure 2B**). The size characterization of TNF antagonists and TNF immune complexes by DLS was carried out in batches over a period of 48 weeks and confirmed the consistency of *in vitro* TNF immune complex formation and analysis (**Supplementary Figure S1**).

### Dendritic cell yields

**Figure 3:**
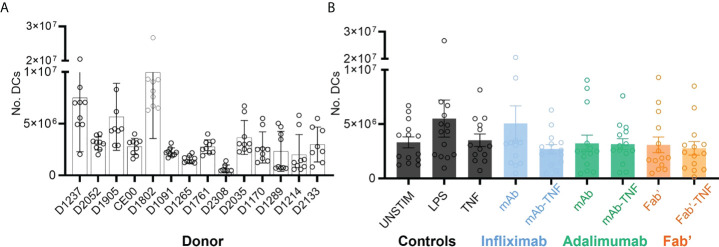
Dendritic cell yields after monocytic purification from PBMCs, LPS stimulation and treatment with TNF, antagonists or immune complexes. **(A)** Total yield of DCs derived from fourteen independent sets of purified CD14+ monocytes. Bars represent the mean and SEM per donor (n=9), where each circle corresponds to one of the nine treatment conditions: UNSTIM, LPS, TNF, INF, ADAL, Fab’, INFL-TNF, ADAL-TNF and Fab’-TNF. **(B)** Yield of DCs plotted by treatment condition: TNF antagonist-free controls (black), INFL and INFL-TNF (blue), ADAL and ADAL-TNF (green) and Fab’ and Fab’-TNF (orange). Each bar represents the mean and SEM per fourteen independently stimulated DCs, where each circle corresponds to the number of cells per donor.

Nine independent sets of DCs were prepared from each of the fourteen donors and on average 3.56 x 10^6^ DCs were obtained per condition. While the yields of DCs were relatively consistent for a given donor, the yields between donor-sets showed significant variability as confirmed by two-way ANOVA (****p < 0.0001, **Figure 3A**). For example, and to illustrate the extremes, D2308 (1.01 x 10^6^) yielded on average a much lower number of DCs compared to D1802 (10.2 x 10^6^). However, average DC yields between treatment conditions were relatively consistent (*p < 0.036, **Figure 3B**).

### Characterization of antigen presentation by HLAII immunopeptidomics

The effect of TNF antagonists and their complexes with TNF on HLAII peptide presentation was investigated next. Across all conditions and donors, on average ~20,000 *MS/MS* spectra matches per immunopeptidome set were recorded, mapping to ~12,000 uniquely identified peptides per condition on average.

**Figure 4:**
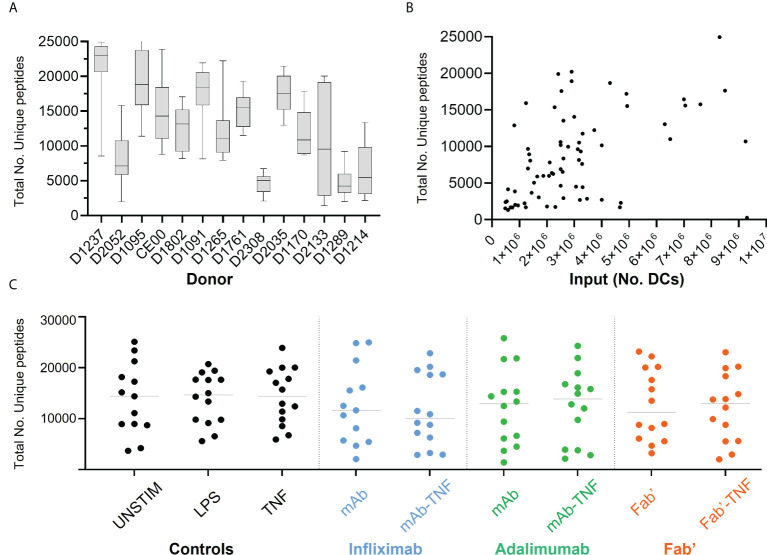
Total HLAII-associated peptides in monocytic-derived DCs. **(A)** Total number of unique peptides identified in the fourteen donors by HLAII immunopeptidomics, each box and whisker plot represent the mean and the SEM of independent data sets per donor. **(B)** Total number of unique peptides plotted against the number of DCs used as input, each point represents an experimental sample (n=124), where samples with >1x10^7^ DCs were not included (2 sets). **(C)** Total number of unique peptides identified in the fourteen donors upon HLAII immunopeptidomics, where each set has the mean and data distribution from fourteen donors per treatment condition corresponding to INFL (blue) ADAL (green) and Fab’ (orange).

Significant inter-donor variability was observed and confirmed by two-way ANOVA analysis (****p < 0.0001, **Figure 4A**). Yields of DCs used for immunoprecipitation correlated weakly with the number of detected unique HLAII peptides (**Figure 4B**). Total number of *unique peptides* were relatively consistent across conditions within any given donor in most of the DC sets (**Figure 4C**). Normalizing the total number of unique peptides per million DCs that were used as input for each analysis did not reduce inter-donor variability and was therefore not pursued further (**Supplementary Figure S2**).

On average, the identified peptides originated from approximately 1,200 protein groups per dataset. Gene ontology analyses of this repertoire in unstimulated DCs compared to LPS-stimulated controls showed an overlap of source proteins with an average and a median of 52% and 55%, respectively, across all DCs (47, 48, **Supplementary Figure S3A**). Overlapping proteins included those involved in mechanisms of HLAII-antigen presentation (i.e., MHC class II protein complex assembly).

However, some source proteins differed between unstimulated and stimulated conditions, including those associated with the regulation of immune-response and antigen presentation processes (**Supplementary Figure S3B**). Source proteins enriched after LPS stimulation corresponded to Fc receptor mediated inhibitory signaling, cell-cell adhesion and regulation of T cell activation *via* T cell receptor contact with antigen. Moreover, in all immunopeptidome datasets the endogenous human protein pool was the main source (> 80%) for HLAII presented peptides independent of DC treatment, followed by bovine proteins from the cell media at ~ ≤20% (**Supplementary Figure S4A**). The peptide presentation of a selected panel of human and bovine source proteins was examined in each sample as quality control. This panel included the intracellular proteins CLIP peptide of invariant chain (CD74) and lysosomal associated membrane proteins (LAMP-1/3); the membrane associated proteins transferrin receptor (TFRC), integrin alpha-M (ITGAM), the immunoglobulin receptors (FCER2/FCGR2A); and the extracellular adhesion protein filamin (FLNA). The quality control panel also included bovine-derived proteins: apolipoprotein B (APOB), hemoglobin fetal subunit beta (HBBF) and albumin (ALB). The detection of at least seven of these control proteins each with a minimum of two unique peptides was required to accept a sample for inclusion in the data analysis. This pre-established acceptance criterion was met by all samples. In most samples, the number of peptides presented from these control proteins remained largely consistent within each donor, however inter-donor variability was evident (**Supplementary Figure S5**).

### Length characterization and binding prediction of HLAII peptides

**Figure 5:**
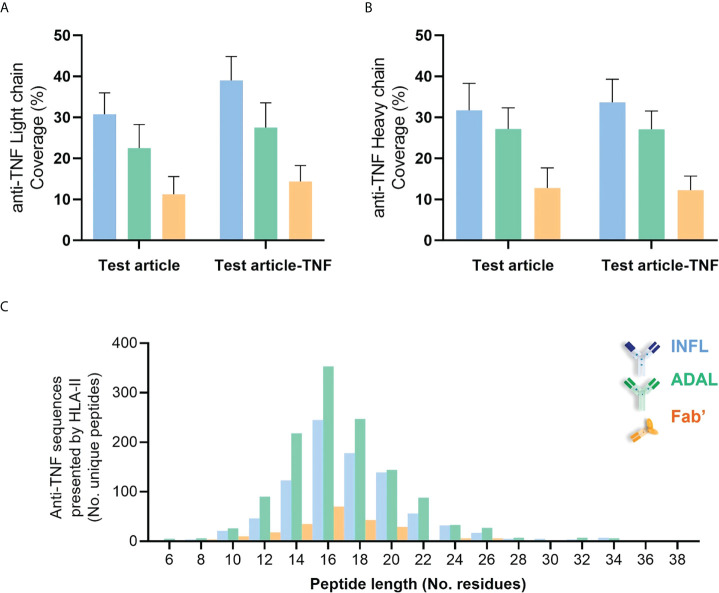
Length distribution of HLAII-associated peptides in monocytic DCs. **(A)** Length of total HLAII immunopeptidome in 126 data sets where each dot represents a detected peptide. **(B)** Frequency distribution of peptide length from a representative donor (D1237), where each bar corresponds to the number of peptides identified at a given residue-length. **(C)** HLAII peptide length distribution from different conditions was analyzed by frequency (D1237). Each bar corresponds to the number of peptides identified at a given residue-length in samples treated with INFL (blue), ADAL (green) and Fab’ (orange) antibodies either alone (*light color*) or TNF-bound (*bold color*).

The peptide length distribution observed in each donor was characteristic for HLAII immunopeptidomes (**Figures 5A, B**). HLAII peptides with 15 amino acids in length were the most frequent across all datasets and peptides with 14-16 amino acids constituting at least 28% of the total immunopeptidome. No significant difference was found in the frequency distribution of the length of HLAII peptides of human or bovine peptides as confirmed by two-way ANOVA (**Supplementary Figures S4B, C**). The consistency of this length distribution was also confirmed across all treatment conditions for test article alone or TNF-bound test article administered to the DCs (**Figure 5C**).

The detection of bovine-derived proteins constituted an opportunity to analyze HLAII presentation of exogenous proteins from the media for their predicted binding strength. Akin to the human peptides, the most frequently detected bovine peptide population was 15 amino acids long. Interrogating these peptides using NetMHCIIpan 4.0 (49) confirmed that the detected bovine peptides were indeed largely predicted as binders with distribution across HLAII alleles per donor set (**Supplementary Figure S6A**). For example, predicted binders mapped to HLA-DR, DP and DQ at 50, ~25 and ~25%, respectively, with or without LPS-induction (**Supplementary Figure S6C**). In addition, the NetMHCIIpan prediction differentiates between strong and weak HLAII binders. For both binder categories, the number of peptides increased by approximately 2-fold upon LPS-induction versus control for all evaluated alleles exemplifying activated HLAII presentation (**Supplementary Figures S6C, D**).

### Characterization of TNF antagonist derived HLAII peptides

**Figure 6:**
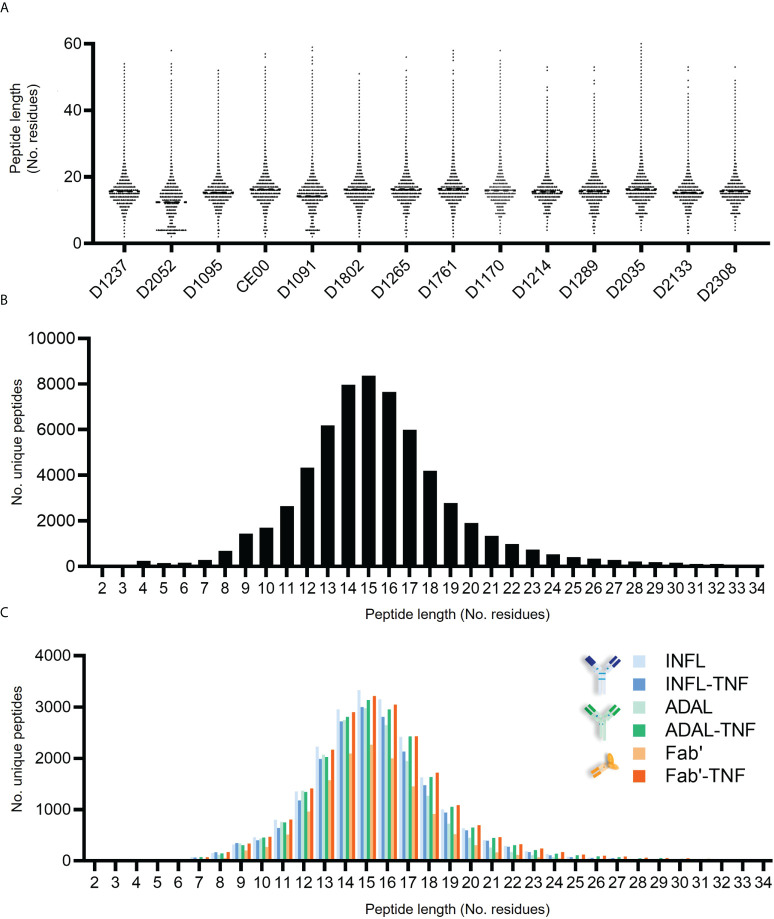
HLAII-presented sequences derived from anti-TNF compounds identified by HLAII immunopeptidomics. **(A, B)** Sequence coverage of identification within both light chain (LC) and heavy chain (HC) from antagonists detected in HLAII immunopeptidomes from antagonist-treated sets corresponding to INFL (blue) ADAL (green) and Fab’ (orange) **(C)** Length distribution of identified TNF antagonist-derived peptides from all donors.

Peptides derived from INFL and ADAL were detected in DCs from all 14 donors, more specifically in 52 of the individual 56 HLAII immunopeptidome sets. The number of peptides presented from both INFL and ADAL was variable across donors (**Supplementary Table 2**), including individual data sets with less than four peptides per sample (e.g., D1289) and those with more than 100 test article-derived peptides per sample (e.g., DCE00 and D1237). INFL peptides were detected at an average of 38 peptides per sample, corresponding to 0.5% of total immunopeptidome and a median of 0.4%. ADAL peptides were detected at an average of 48 peptides per sample, corresponding to approximately 0.5% of the immunopeptidome (average and median). In contrast, Fab’-derived peptides were detected in 12 donors (18/28 HLAII immunopeptidome sets) with an average of 10 peptides per sample, corresponding to an average and a median of 0.2% and 0.06% of total immunopeptidome (**Supplementary Table 2**). In agreement with these findings, the detected protein sequence coverage in both light and heavy chains was generally higher from INFL and ADAL-treated DC compared to Fab’ treatment in most donors (**Figures 6A, B** and **Supplementary Figure S7**). Frequency distribution of length of HLAII peptides from all three TNF antagonists were concordant with the global HLAII immunopeptidome (**Figure 6C**).

### Influence of TNF-antagonist complexes with TNF on TNF-antagonist peptide presentation

**Figure 7:**
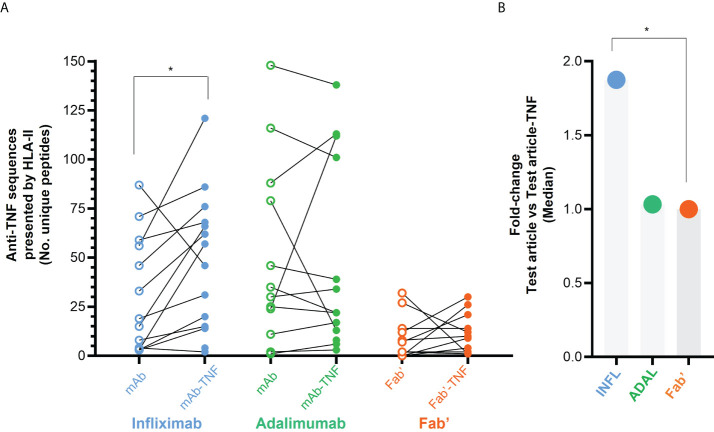
HLAII-presented sequences derived from anti-TNF biotherapeutics. **(A)** Number of unique anti-TNF peptides derived from DCs loaded with test article alone (empty circles) or in complex with TNF (full circles) per donor, corresponding to INFL (blue), ADAL (green) and Fab’ (orange); (*p < 0.013 by Wilcoxon marched-pairs signed rank test) **(B)** Median fold-change anti-TNF *vs.* anti-TNF complexed with TNF based on the number of unique anti-TNF peptides detected per sample. ns, non-significant; *p > 0.036 by Mann-Whitney analysis.

Unique sequences derived from INFL were variable across donors but were found to increase 1.2- to 19-fold in most INFL-TNF immunopeptidomes compared to the INFL condition (10 of the 14 donors; **Figure 7A**; arranged by condition in **Supplementary Figure S8A** or arranged by donor **Supplementary Figure S9**). A relatively consistent number of INFL peptides was detected between INFL and INFL-TNF in only one donor. An approximately 2-fold decrease in unique INFL peptides in the INFL-TNF condition relative to the INFL alone was observed in two donors. Across all donors, a median of 1.87-fold increase in INFL derived sequences was detected in the INFL-TNF condition, which was statistically significant relative to the Fab’ (**Figure 7B**).

The presentation of unique ADAL sequences was also variable across donors (**Figure 7A**; arranged by condition in **Supplementary Figure S8A**). In five of the 14 donors the number of ADAL sequences increased at 1.2- to 6-fold while two donors had no test article-peptides in the ADAL condition but detectable peptide in the ADAL-TNF condition. A relatively consistent number of ADAL peptides between ADAL and ADAL-TNF conditions was detected in five donors. A 1.5- to 6-fold decrease in unique ADAL peptides in ADAL-TNF immunopeptidomes relative to ADAL was observed in two donors. The average remained unchanged between ADAL and ADAL-TNF condition (**Figure 7B**).

Similarly, the single arm Fab’-TNF treatment resulted in an unchanged average presentation of Fab’ derived HLAII peptides between Fab’ and Fab’-TNF conditions (**Figure 7B** **;** **Supplementary Figure S8A**). In three donors a 1.2- to 3-fold increase in Fab’ peptide presentation was detected with Fab’-TNF complexes while there was no change in two donors. A ~ 2- to 32-fold decrease in unique Fab’ peptides in the Fab’-TNF condition relative to the Fab’ alone was observed in three donors. No Fab’ peptides were detected in either condition in the remaining donors.

A subsequent investigation focused on identification of sequences that, despite the donor-to-donor variability, were responsible for the change in test article presentation in the TNF immune complex condition for INFL (**Figure 7B**). To that end, an increase in the presentation of light chain (LC) peptides was observed between INFL and INFL-TNF treatment, but not for ADAL or Fab’. The number of heavy chain (HC) peptides being presented between the three TNF antagonists and their TNF immune complexes appeared to be consistent (**Supplementary Figures S8B, C**). In general, the HLAII presented peptides originating from INFL, ADAL and Fab’ proteins, mapped not only to constant but also to a lesser degree to variable regions from both heavy- and light-chains (VH and VL). For example, more than 10 DC sets treated with INFL or INFL-TNF presented peptides spanning VL3-20 (**Supplementary Figure S10A**). A similar case was observed in at least five donors, where prominent nested sets with more than five unique peptides included sequences spanning VL178-197 from INFL, ADAL and Fab’ and their TNF complexes (**Supplementary Figure S10A**). This predominant presentation of framework regions was also the case for VH70-90 from the three test articles (**Supplementary Figure S10B**).

### Detection of CDR sequences derived from TNF antagonists

HLAII peptides fully or partially spanning CDRs from the three TNF antagonists without TNF immune complexes were identified in 7, 9, and two donors for INFL, ADAL, and Fab’, respectively (**Table 1**). Sequences covering the LCDR1 of INFL and ADAL were only observed in one donor each, while Fab’-LCDR1 was not detected. In contrast, LCDR2 was detected mainly in INFL and ADAL in four donors each, but also in three donors treated with Fab’. Only one and three donors for INFL and ADAL, respectively, presented peptides spanning the LCDR3. Peptides including INFL- and ADAL-HCDR1 were identified in 2 and 1 donors each, while no donors presented peptides spanning the Fab’-HCDR1. INFL- and ADAL-HCDR2 was presented more prominently in seven and six donors, respectively. This was also the case of the HCDR3 presented in six, four, and one donors for INFL, ADAL, and Fab’.

**Table 1:** CDR sequences from TNF antagonists presented by the HLAII.

	INFL	INFL-TNF	ADAL	ADAL-TNF	Fab’	Fab’-TNF
**(A) Number of donors presenting CDR sequences**	7	11	9	7	2	4
**(B) Total number of detected unique CDR peptides from all donors/all test article peptides**	104/407	148/668	165/605	140/645	5/106	28/131
**(C) Normalized response of CDR/all test article peptides from all donors (dataMAPPS)**	785/3920	968/5776	882/4353	889/4385	32/556	89/514

Presentation of CDR peptides changed in some of the DCs treated with TNF-complexes compared with TNF antagonist controls. The most notable change was for INFL with a ~1.5-fold increase in the *number of donors* and *number of unique* INFL and CDR peptides identified with INFL-TNF treatment (**Tables 1A, B**). From the 11 donors presenting CDR peptides with INFL-TNF treatment, eight were higher and three were lower presenters relative to INFL treatment. While most of the LC-peptides presented from INFL mapped to the germline sequences independent of treatment (INFL *vs.* INFL-TNF), the INFL-LCDR2-sequences were only detected in two donors (D1170 and D1214) treated with INFL-TNF. Subtle differences in presentation of LCDR1 and LCDR3 were detected in D1802, D1761 and D2035, where a partial coverage (1-3 residues) was found in TNF immune complex treated DCs. Peptides covering HCDR2 and HCDR3 increased in INFL-TNF in three donors (DCE00, D1237 and D1214). The three cases where CDR-presentation decreased upon TNF immune complex treatment included sequences spanning HCDR2 and HCDR3 in D1095, HCDR3 and LCDR2 in D1901, HCDR3 in 1802 and HCDR2 in D1289.

In order to further characterize CDR-presentation, immunopeptidomes were normalized based on peptide *peak area* using *dataMAPPs* (37). Upon normalization, HLAII presentation of nested sets (clusters) of hypervariable regions spanning CDR-sequences showed different patterns across donors in response to antagonists and immune complexes (**Figure Supplementary S11** **)**. Of note, increase in INFL-presentation in DCs treated with INFL-TNF based on peptide counting was concordant with the increase observed in the GRSN-normalized response of peak area from INFL peptides as well as those detected spanning the CDRs (**Table 1C**; **Figure Supplementary S12** and **S13**). A 1.2- and 1.5-fold increase for all INFL peptides and INLF-CDR peptides, respectively, was calculated by the sum of normalized signal intensity upon GRSN (log2).

Peptides derived from ADAL-CDRs in response to TNF immune complexes also varied intra- and inter-donor with a ~1.3-fold decrease in the *number of donors* and overall, a consistent *number of unique* ADAL and CDR peptides identified with ADAL-TNF treatment (**Tables 1A**, **B**). From the seven donors presenting CDR peptides with ADAL-TNF treatment, four were higher, two were lower presenters and one remained constant relative to ADAL treatment. For the first group of higher presenters, HCDR1 and LCDR1 were detected only in ADAL-TNF for donor DCE00, where HCDR2 increased and HCDR3 and LCDR2 remained unchanged relative to ADAL. For donor D1802, HCDR3 and LCDR3 increased in ADAL-TNF, while HCDR2 and LCDR1 remained consistent to ADAL. For donor D1237, only LCDR2-peptides increased in ADAL-TNF compared to ADA, while LCDR3 and HCDR3 were found unchanged. D1901 showed increased presentation of HCDR2 and LCDR3. The second group of lower presenters included donor D1265, which showed a partial coverage (5/9 residues) of HCDR2 only in ADAL-induced DCs and a decrease in LCDR2-presentation in ADALTNF. In donor D1095, presentation of HCDR2 was also reduced in ADAL-TNF compared to ADAL.

Lastly, relatively consistent presentation of HCDR2, HCDR3 and LCDR2 characterized both donors D1265 and D2308. Although the listed responses upon ADAL-TNF were not completely recapitulated upon normalization by GRSN for donors with low number of peptides (D1095, D2308 and D1901) as well as for sequences with low presentation (≤2 peptides), such as LCDR1 and HCDR1 for ADAL-TNF in DCE00 (**Figure Supplementary S11** and **S13**), overall ADAL presentation was not affected by TNF complexes with no substantial changes in normalized response of total and CDR-peptides (**Table 1C**).

CDR presentation from Fab’ was the lowest amongst the three TNF antagonists and also varied intra- and inter-donor. Even though, a 2-fold increase in the *number of donors* and a 1.2- to 5.6-fold increase in *number of unique* Fab*’* and Fab’-CDR peptides was identified with Fab’-TNF treatment (**Tables 1A, B**), these changes are not considered quantitively reliable or representative due to the low and variable presentation of Fab’ and Fab’-CDR peptides. Most of the donors did not present CDR peptides in Fab’ treatment (D2133, D1265, D1761, D1095 and D1901). Only D1802 showed an increased HCDR2 and LCDR2-presentation in Fab’-TNF, corresponding to 3.5-fold compared to Fab’ treatment. In contrast, donor D1237 showed a decrease of LCDR2 in Fab’-TNF samples compared to Fab’. HLAII-peptides extending 4-5 residues into the HCDR2 were detected in three donors, where only one of them showed presentation exclusively in Fab’-TNF upon GRSN normalization. Fab’-LCDR2 and LCDR3 peptides were exclusively found in Fab’-TNF sets in two and one donors, respectively.

## Discussion

In this study, we examined the effect of antibody-target complex formation on the presentation of therapeutic antibody derived peptides in an *ex vivo* system which models natural antigen processing and presentation. Antagonists INFL, ADAL and Fab’ were compared alone or in complex with TNF.

Overall, measurements of size of the TNF antagonists alone were consistent with previous reports based on DLS and other biophysical assessments (50–55). Of note, the distinct difference in size observed for the bivalent antibodies ADAL and INFL (10.5 ± 1.0 nm and 17.5 ± 2.4 nm, respectively), despite their equivalent molecular weight (MW, 56), may result from differential physicochemical properties as previously suggested (50–55, 57–60). Reversible self-association of INFL has been associated with an apparent size increase (61, 62). Despite the smaller MW in the case of Fab’, PEGylation has been estimated to increase the size ~ 3 to 5-fold relative to the non-conjugated molecule (63).

We observed that TNF immune complexes that were formed *in vitro* were larger in size for INFL relative to ADAL and Fab’, consistent with previous studies (46, 50–52, 57–59). These differences can be explained by the structural conformation and binding dynamics between the different antagonists and TNF. Trimeric TNF possesses three binding sites for INFL and ADAL antibodies, each having two TNF binding sites, favoring the formation of immune complexes with various binding stoichiometries (50–52, 57–59). For example, a single Fab’ arm of an INFL molecule can bind to one monomer of the TNF homotrimer (57), hence, each INFL molecule can bind to two TNF homotrimers, which allows the possibility of antibody-target complex formation. Furthermore, formation of high-molecular weight structures is favored due to the ability of three INFL molecules to bind a single TNF homotrimer, leaving 3 free antibody binding sites available for further complex formation. In addition, conformational changes induced by INFL binding to TNF are suggested to stabilize the TNF homotrimer, eliminating monomer exchange. Indeed, the TNF complexes formed with INFL-TNF were significantly larger, i.e., ~70 nm, compared to the other two compounds in this study. In contrast, each Fab’ arm of an ADAL molecule binds between two monomers of the TNF trimer, which also leaves the second Fab’ free for the formation of complexes (57), however, ADAL formed smaller complexes relative to INFL, i.e., ~40 nm. The difference in size of TNF complexes between INFL and ADAL may be explained by their different potential of stabilizing the trimeric TNF depending on antigen concentrations, which increase the propensity of monomeric exchange (53–55). Finally, only a minor size increase was noted upon TNF-binding with Fab’ (~20 nm). This is consistent with monovalent TNF antagonists, which cannot form complexes that incorporate more than one TNF as well as their inability to stabilize TNF as a homotrimer (50, 55).

While stimulation with TNF antagonists alone or in complex with TNF did not affect the yield of DCs, yields were highly variable between donors. The total number of unique HLAII peptides detected per donor was also variable, however, no strong correlation was found with the number of DCs used as an input for HLAII immunopurification (**Figure 4B**). Furthermore, the presentation of test article-derived peptides from INFL, ADAL and Fab’ varied independently from the total number of HLAII peptides detected in each sample. For example, while DCE00 presented ~9,000 HLAII peptides in total and, on average, 0.85% of the total immunopeptidome was derived from TNF antagonists, donor D1237 presented on average ~16,000 unique total peptides, with 0.42% of the total immunopeptidome coming from TNF antagonist. These observations suggest that other factors, such as the inherent characteristics of the antigenic sequences as well as the donor HLAII-haplotype are more likely the key determinants for presentation of TNF antagonist sequences. In addition, factors important to the regulation of peptide-presentation are likely to play a role as well, such as the abundance of the HLAII protein, which has been shown to be donor dependent (39).

Given the variability in the number of unique peptides in the immunopeptidome across donors and DC yields, the HLAII presentation of INFL, ADAL and Fab’ was also found to be variable. Furthermore, the bivalent antibodies were predominantly detected in all donors consistent with their detection in prior studies (32–34). In contrast, the Fab’ anti-TNF was detected in only a little more than half of the donors (8/14 donors; **Figure 7**, **Supplementary Figure S8**). Normalization of all detected peptides in an immunopeptidome by their *peak area* within and between donors, by *dataMAPPs* analysis (37), confirmed a wide range of test article presentation depending on the antagonist and donor, where increased presentation of INFL and ADAL compared to Fab’ was confirmed.

HLAII-peptides derived from INFL included variable regions in both the heavy and light chain (VH and VL). For example, the framework region between HCDR2 and HCDR3, VH71-94, was consistently detected in 50-57% of DC sets analyzed in prior and current investigations (17/34 and 8/14 donor-sets, respectively; 36-37). Furthermore, peptides from the light chain region, VL3-19, were presented in 50% of the DCs (7/14 donors). HLAII-presentation of hypervariable regions included HCDR2, HCDR3 and LCDR2 sequences in 64, 42 and 28% donor-sets, respectively, consistent with previous studies (33, 34). In addition, new peptides derived from INFL were discovered, which overlapped LCDR1 and HCDR1 regions, at least partially, in 3/14 donors (D1801, D1170 and D1901). These novel INFL sequences may be immunogenically relevant given the overlap with T-cell activation epitopes in patients with anti-drug antibody responses (33). This is also the case of LCDR3 sequences detected at 28% (4/14 donor sets) in the present study.

ADAL-presentation also aligned with prior immunopeptidomics work (32), including HLAII-peptides with T-cell activation potential spanning the LCDR2, LCDR3 and HCDR3 (~ 20-50% of donor-sets). Presentation of ADAL peptides partially spanning HCDR1 were found at in 28% of the donors (4/14 donor-sets). As for INFL, novel HLAII-peptides from ADAL were identified, such as multiple sequences including VL71-85 in at least 35% of donor-sets (5/14). Peptide sequences including VH3-20 and VL120-128/126 as well as the VL1-15 from ADAL were detected bound to the HLAII at a lower degree (2-3/14 donor-sets), where a peptide discovered spanning 4/6 residues into LCDR1 is perhaps more immunogenically relevant (D1265). In summary, the HLAII-presentation of hypervariable regions from both INFL and ADAL, showed HCDR1 and LCDR1 as the lowest presented in contrast to HCDR2, HCDR3, LCDR3 and LCDR2, in agreement with prior investigations.

Furthermore, the influence of TNF immune complexes on peptide presentation was clear for INFL, increasing presentation of test article-derived peptides including hypervariable regions relative to untreated INFL in most donors. Specifically, the number of positive donors and *unique peptides* presented from CDR-sequences increased in INFL-TNF, which was confirmed not only by peptide counting but also by analysis of normalized peak area using *dataMAPPs* (**Table 1**). Of note, INFL forms the largest high-order structures with TNF of the compounds tested. This correlation between the size of TNF immune complexes and increased INFL presentation is similar to the increased HLAII-presentation observed in aggregating mAbs (40, 41), which was attributed to the higher effective test article amount available to the intracellular processing machinery upon the internalization of densely compacted proteinaceous particles by APCs. Aligned with these findings, aggregated INFL by heat stress significantly induces DC activation in contrast to ADAL and other bivalent antibodies (42). However, questions remain about the potential effects on protein uptake, processing, and presentation in DCs by of high-molecular weight structures. For example, internalization of INFL-TNF immune complexes was shown to occur independently of FcγR in DCs (45), where the increased number of INFL molecules present in a single internalized immune complex with TNF relative to INFL alone may explain the increased relative quantity of epitopes for processing and presentation of T-cell epitopes (40). Furthermore, the increase in peptide presentation following complex formation was not observed for ADAL, which formed smaller complexes with TNF compared to INFL. In fact, the opposite effect was seen in several donors, for which test article presentation decreased upon immune complex formation with TNF. This may suggest that TNF immune complexes do not influence the presentation of the humanized ADAL, which was highly presented in the absence of immune complexes. The effect of TNF-immune complexes with Fab’ remains unclear because overall presentation of Fab’ was detected in only a few donors, generally with less than four peptides. Even though only a few donors presented Fab’ peptides, an increase of CDR-presentation in response to TNF complex was observed in most of them. The contribution of the smaller size of the Fab’ molecule to its presentation cannot be ruled out given the lower probability of epitope generation relative to larger sized antagonists such as INFL and ADAL. In addition, the low presentation of Fab’ may also be compounded by its conjugation to the polyethylene glycol (PEG) group. Indeed, a comparison of a single-armed anti-TNF Fab’ with and without PEG showed differences of internalization by DCs as well as T-cell elicited response, being both significantly lower for the pegylated Fab’ (64).

To our knowledge, this investigation provides the first evidence of the effect of INFL-TNF immune complexes on HLAII-peptide presentation, suggesting that high-molecular weight drug-target structures may amplify the immunogenic response in certain individuals. In agreement with this hypothesis, INFL-TNF complexes increase the anti-drug antibodies (ADA)-response *in vivo* (45, 46). This was also observed for another TNF antagonist: a bi-specific antibody targeting TNF and TL1A, another trimeric protein of the same family. Immune complexes with this bi-specific antibody were associated with higher ADA response in 53/54 healthy donors administered with the compound (65). Thus, TNF concentrations, compounded by the individual genetic- and proteomic-background, may affect HLAII-levels of presentation upon formation of immune complexes with antagonists. TNF levels in subjects under treatment with INFL and ADAL increase after 7-days, showing a significant inter-donor variability (44, 66). Of note, all ADAL is bound to TNF (ADAL-TNF), which levels are inversely correlated with ADA response in a percentage of individuals (44). This correlation suggests the potential relevance of TNF-immune complexes preceding ADA.

Quality controls and quantitative parameters to describe HLAII-presentation (i.e., definition of *peptide-clusters* based on number of *unique peptides*, *nested sets* and *peptide abundance)* continue to be established. For example, intra- and inter-donor normalization strategies, which are crucial for the comprehensive assessment of HLAII-presentation and relative quantitative interpretation of immunopeptidomics studies, are still being explored. Potentially, peptides presented from proteins internalized from the media such as APOB, HBB, ALBUM could serve as quality controls in HLAII-studies or as a tool to normalize the signal of HLAII-presented peptides. To this end, the generation of a master pool of DCs stimulated with a qualified standard HLAII-presented protein under same conditions as the test article may provide a valuable tool for quality assessment in HLAII immunopeptidomics. Quantitative side-by-side comparisons were not feasible given the known differences in donors analyzed and sample preparation workflows employed (67), however our findings shed light on the robustness of HLAII immunopeptidomics to investigate-presentation of biotherapeutics, as similar degree of HLAII-presentation for TNF antagonists were found across studies despite significant differences in instrumentation and analytical approaches.

## Conclusion

Altogether, our results suggest that increased CD4+ T cell epitope presentation of drugs when in complex with their target could play a role in the correlation that has been described between clinical immunogenicity of TNF antagonists and immune complex formation. Alongside with HLAII peptide presentation, T-cell response is higher for INFL, followed by ADAL and Fab’. Hence, we hypothesize that the wide immunogenicity rates and predominance of INFL presentation in contrast to the ADAL and Fab’ aligns with the formation of high-ordered structures with TNF, which have been recently detected in patients with bivalent antagonists (44–46). However, HLA II peptide presentation does not represent an immunogenic response *per se*. Therefore, further studies with anti-TNF-TNF immune complexes and other drug-target complexes at the HLA-allele specific level, including new modalities, as well as the corresponding T-cell activation responses will contribute to the determination whether high molecular weight drug-target complex formation constitutes an immunogenicity risk factor.

## Data availability statement

The original contributions presented in the study are publicly available. The data presented in the study are deposited in the MassIVE repository. Datasets are accessible with the following link: https://ftp://massive.ucsd.edu/MSV000090169/.

## Ethics statement

The commercially acquired PBMCs from human participants were collected by STEMCELL Technologies (Vancouver, BC, Canada) under the review and approval of an Institutional Review Board (IRB). This IRB is in full compliance with good clinical practices as defined under the U.S. food and drug administration (FDA) regulations, U.S. department of health and human services (HHS) regulations, and the international conference on harmonisation (ICH) guidelines. The participants provided their written informed consent that their samples can be used in biomedical research.

## Author contributions

AC-L and HN designed the study. AC-L acquired data by performing most laboratory experiments and wrote the manuscript; HN, RS, TH, ST, and AC-L conceptualized and developed the workflow for HLAII-immunopeptidomics. MW and GK enabled and performed the LCMS analysis of the DC-datasets by timsTOF MS. MA developed *in silico* tools for analysis. MA and GB carried out *in silico* analysis for peptide normalization to input material. AC, NA-A, and GB assisted with sample preparation. ZL and H-YK enabled DLS analyses. HN supervised the work. All authors critically revised the paper for intellectual content. All authors contributed to the article and approved the submitted version.

## Acknowledgments

This work has been supported by the Pfizer Worldwide Research, Development and Medical Postdoctoral program.

## Conflict of interest

AC, GB, ZL, H-YK, HN, RS, ST, and AC-L are current employees of Pfizer Inc. MA, NA-A, and TH were employed at Pfizer when they contributed to this study. GK and MW are current employees of Bruker Daltonics.

## Publisher’s note

All claims expressed in this article are solely those of the authors and do not necessarily represent those of their affiliated organizations, or those of the publisher, the editors and the reviewers. Any product that may be evaluated in this article, or claim that may be made by its manufacturer, is not guaranteed or endorsed by the publisher.
