# Supplementary material for: HLAII peptide presentation of infliximab increases when complexed with TNF
Source: Front Immunol. 2022 Sep 13;13:932252. doi: 10.3389/fimmu.2022.932252 (PMC9513746; doi:10.3389/fimmu.2022.932252)
Supplement: Supplementary file 1 [file DataSheet_1.pdf]

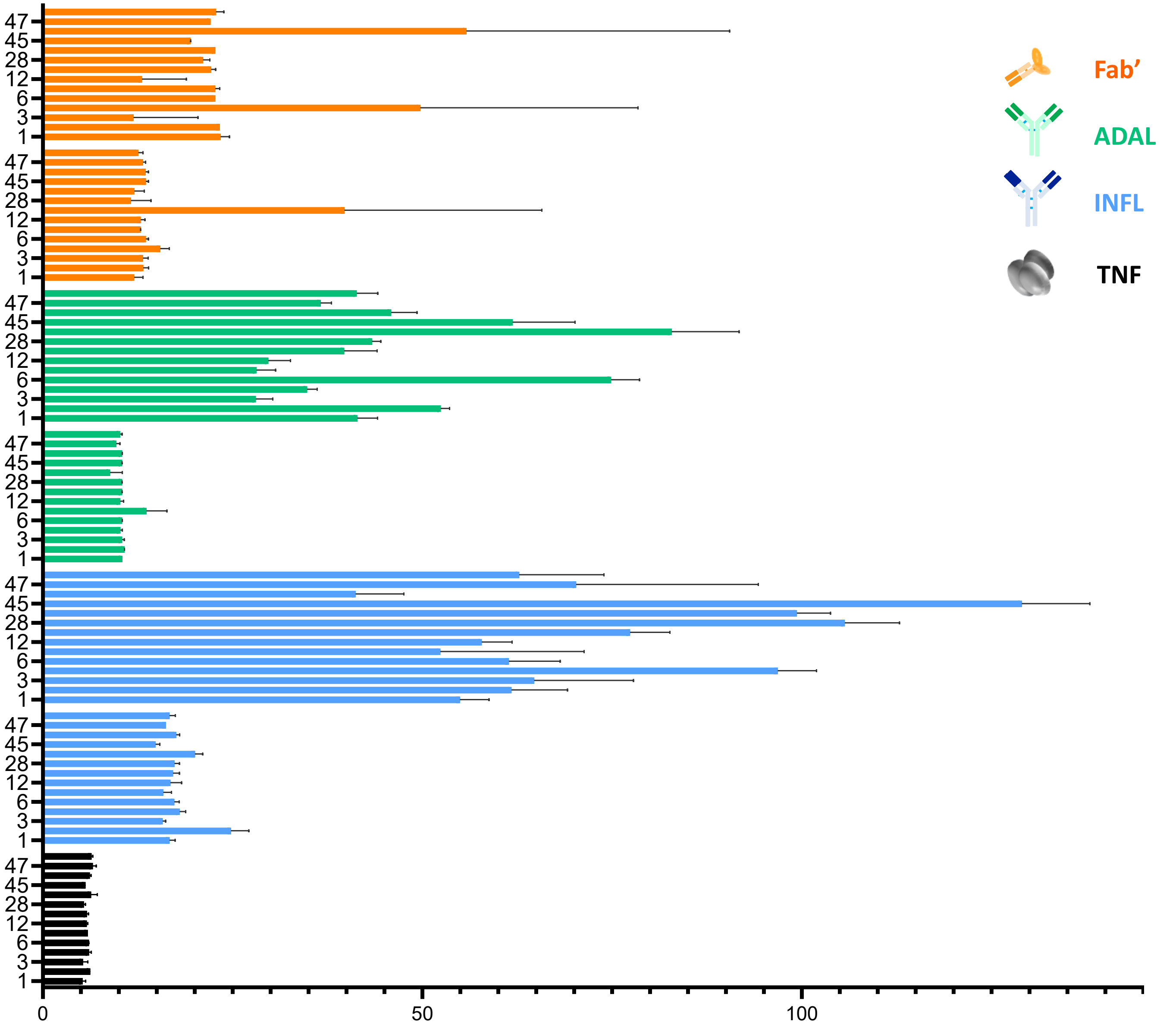

Supplementary Figure S1. Size characterization of TNF-immune complexes formed during 48 weeks of experimentation. *In vitro* complex formation was monitored along the study in terms of size by DLS. Sets of antagonists alone (bottom) or in complex with TNF (top) are presented. Each bar corresponds to the mean of three replicates and the SEM from independent experiments (n=14).

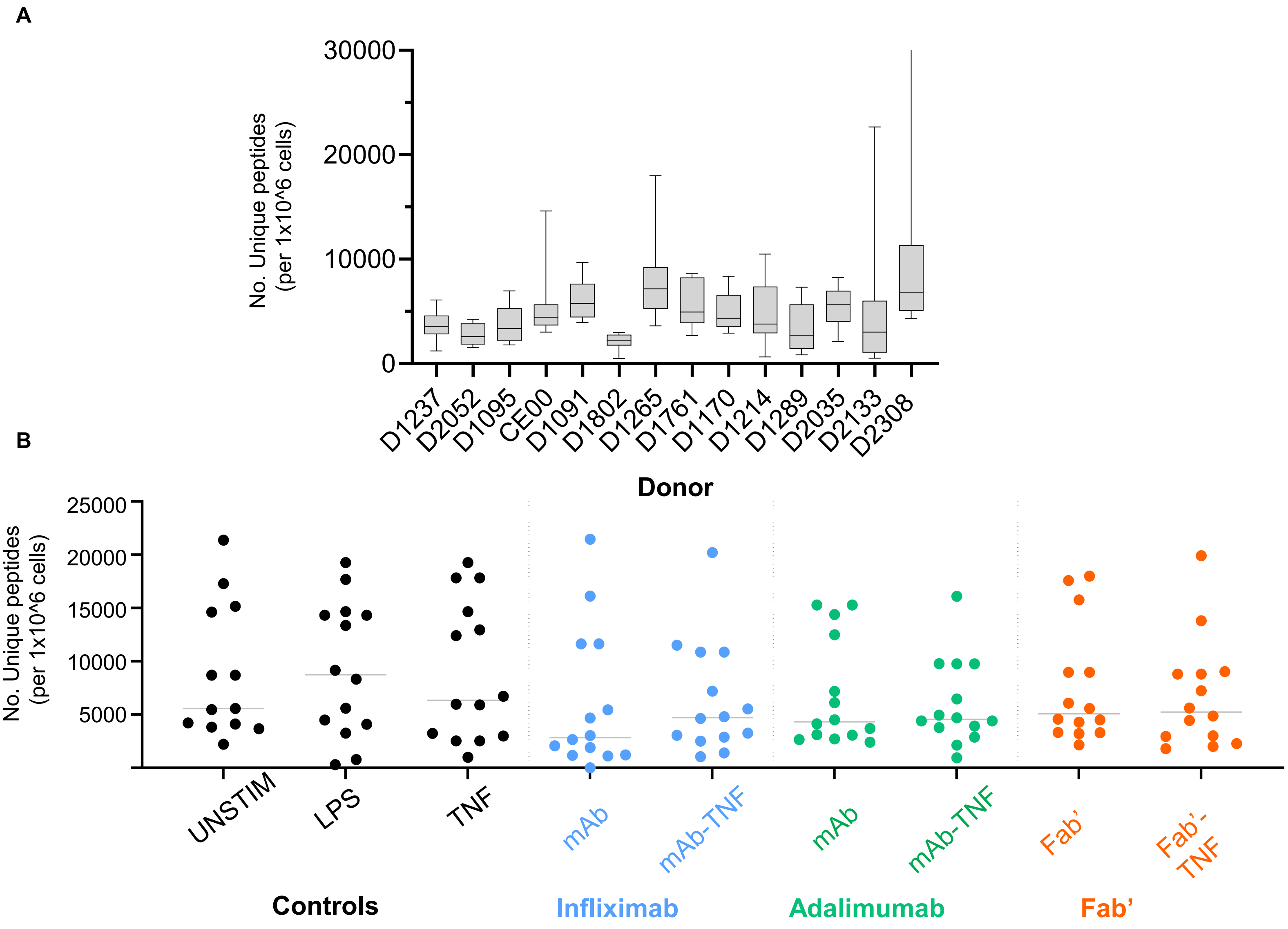

Figure S2. Total HLAII-associated peptides in monocytic-derived DCs detected by timsTOF normalized to input DCs. Total number of unique HLAII-associated peptides identified after DB-search per million of DCs separated by donor in box and whisker plots **A** or treatment condition **B**, where each replicate and the mean is presented (n=14).

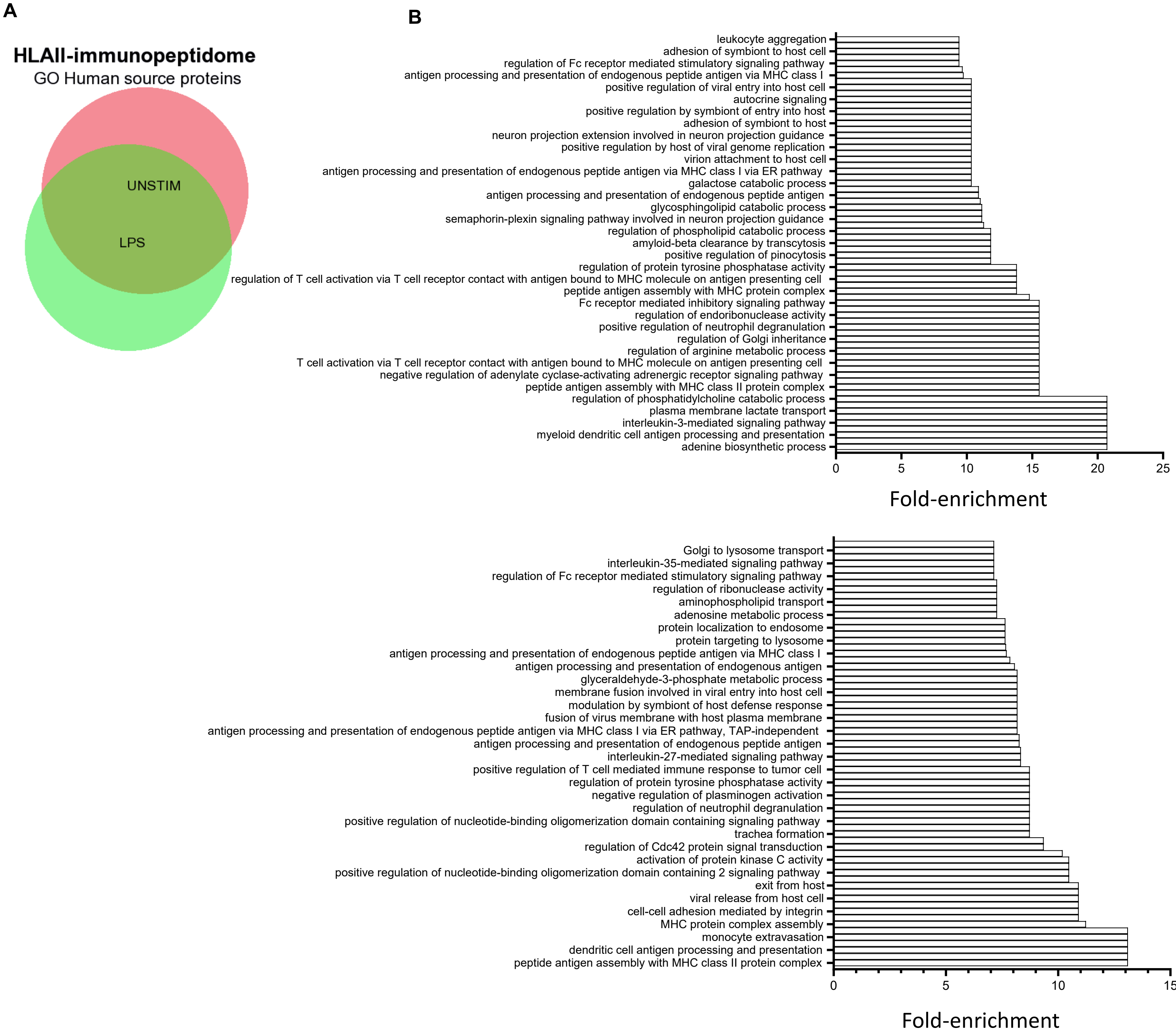

Supplementary Figure S3. GO Human-derived peptides presented by the HLAII on monocytic DCs. A. Ven diagram of the number of HLAII-peptides detected from the human proteome in LPS-induced cells (Green) vs UNSTIM control (RED) , **B** Biological function categories identified by Fisher exact test from UNSTIM control (Top panel) and LPS-induced cells (Bottom panel).

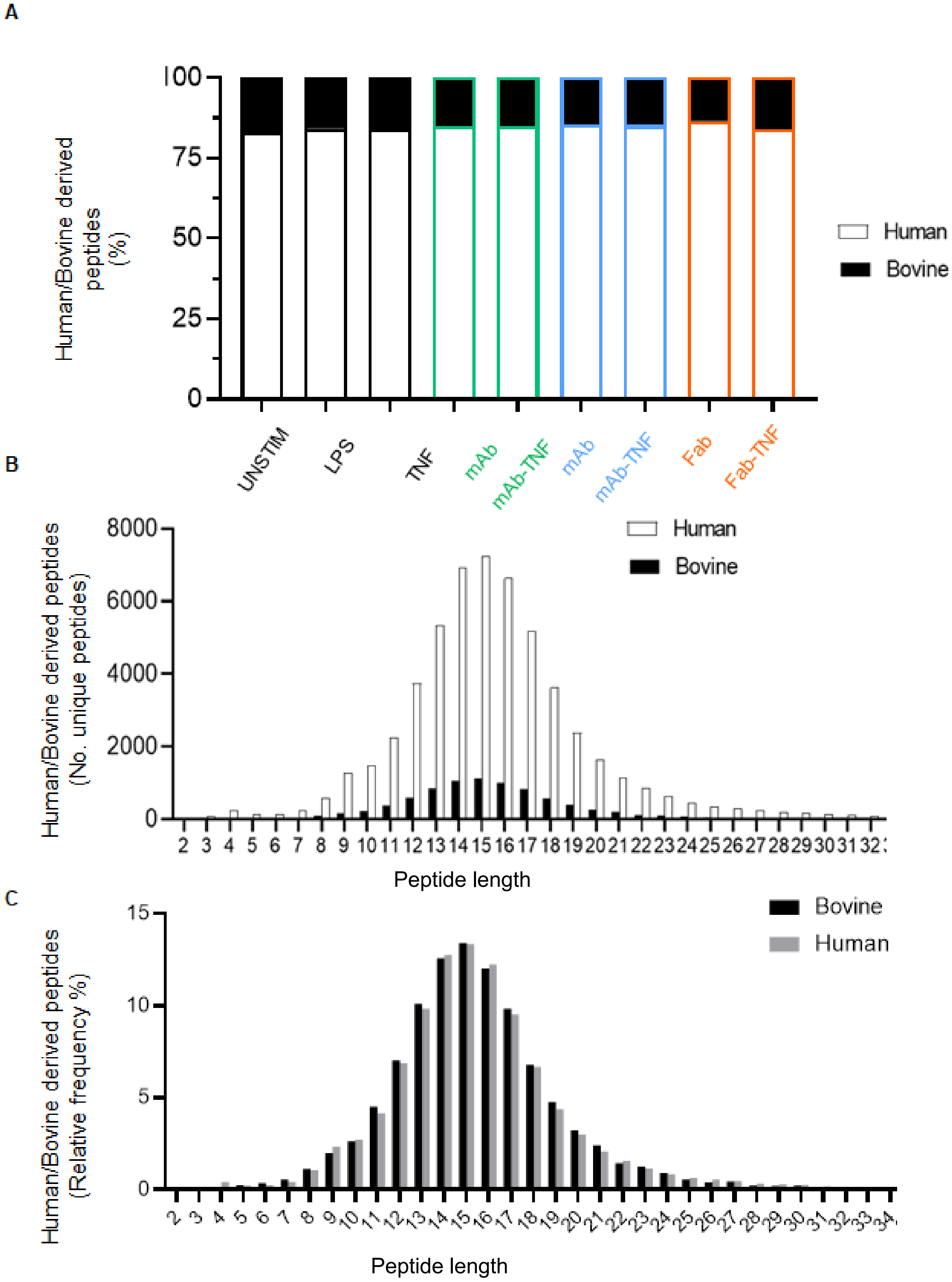

Supplementary Figure S4. Bovine- and human-derived peptides presented by the HLAII on monocytic DCs. (A) Source proteins of HLAII-peptides detected in DCs of a representative donor. Each bar represents the mean and SEM of nine datasets. **(B)** Percentage of peptides identified from the bovine and human proteome was analyzed in each treatment condition, including controls UNSTIM LPS and TNF as well as INFL (Green), ADA (blue) and Fab' (Orange). **(C)** Length distribution of both human and bovine peptide-populations was analyzed by frequency. (**D)** Frequency distribution of bovine- and human- derived peptides in terms of relative frequency (percentage).

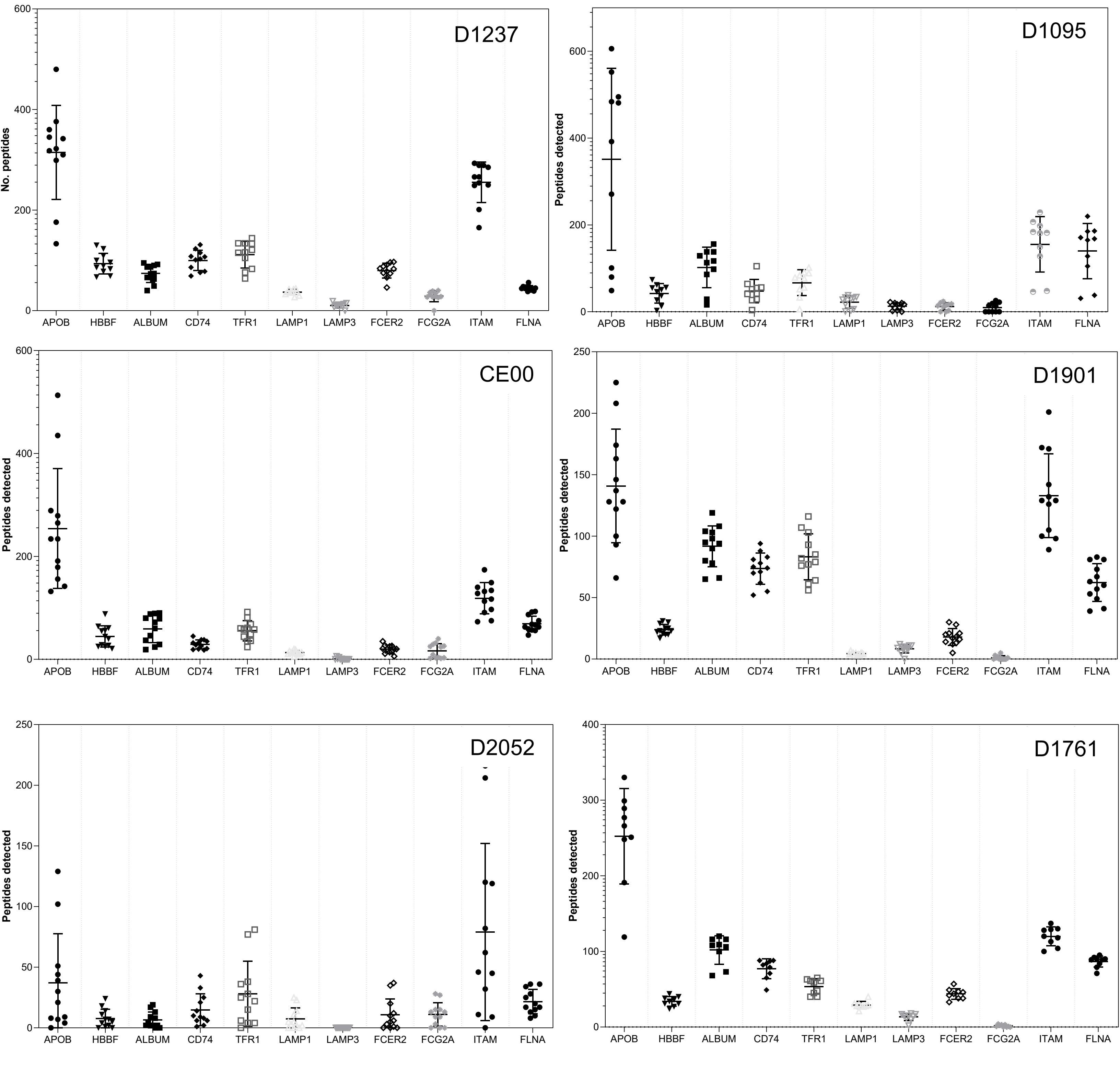

Supplementary Figure S5. Analysis of quality control proteins presented by the HLAII on monocytic DCs of six representative donors. The number of unique peptides derived from a set of three bovine proteins and eight endogenous proteins is shown. The mean and SEM is presented per protein, where each point corresponds to the number of unique peptides identified for treatment condition of DCs.

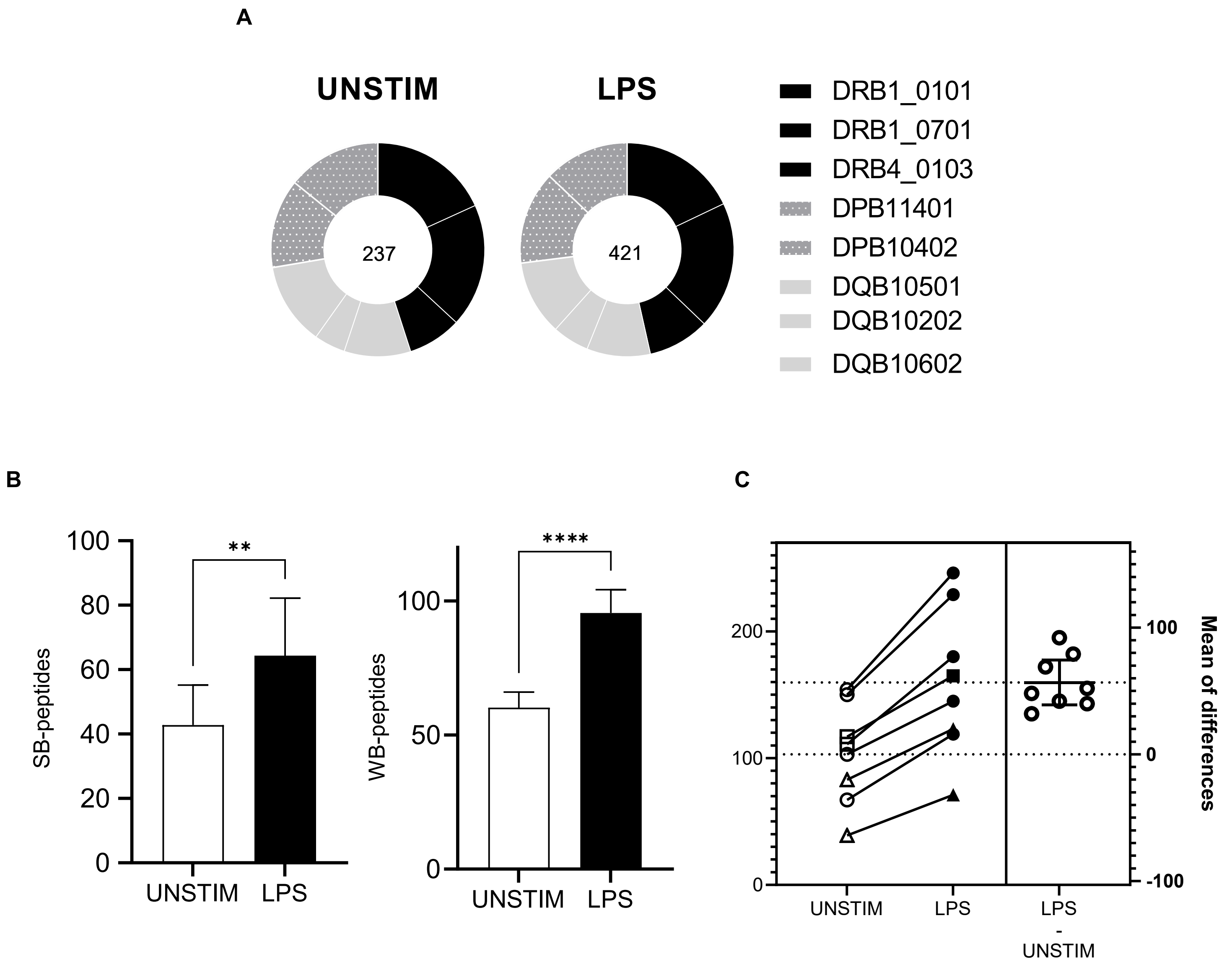

Supplementary Figure S6. Analysis of Bovine-peptides presented by the HLAII on monocytic DCs of a representative donor (D1237). (A) Predicted pattern of binding to the HLAII-haplotypes based on binding affinity and eluted peptides by NetMHCII4.0. **(B)** Number of strong- and weak-binders (SB and WB, respectively) predicted by NetMHCII4.0 in UNSTIM and LPS controls (**p< 0.0095 and **p<0.0001 by two tailed T-test). **(C)** Increase in number of peptides predicted to bind to HLAII by NetMHCIIpan4.0; each point representing the number of predicted binders per haplotype in the UNSTIM (empty) control and after LPS (black). Significance of difference is expressed as the mean on far right (LPS – UNST).

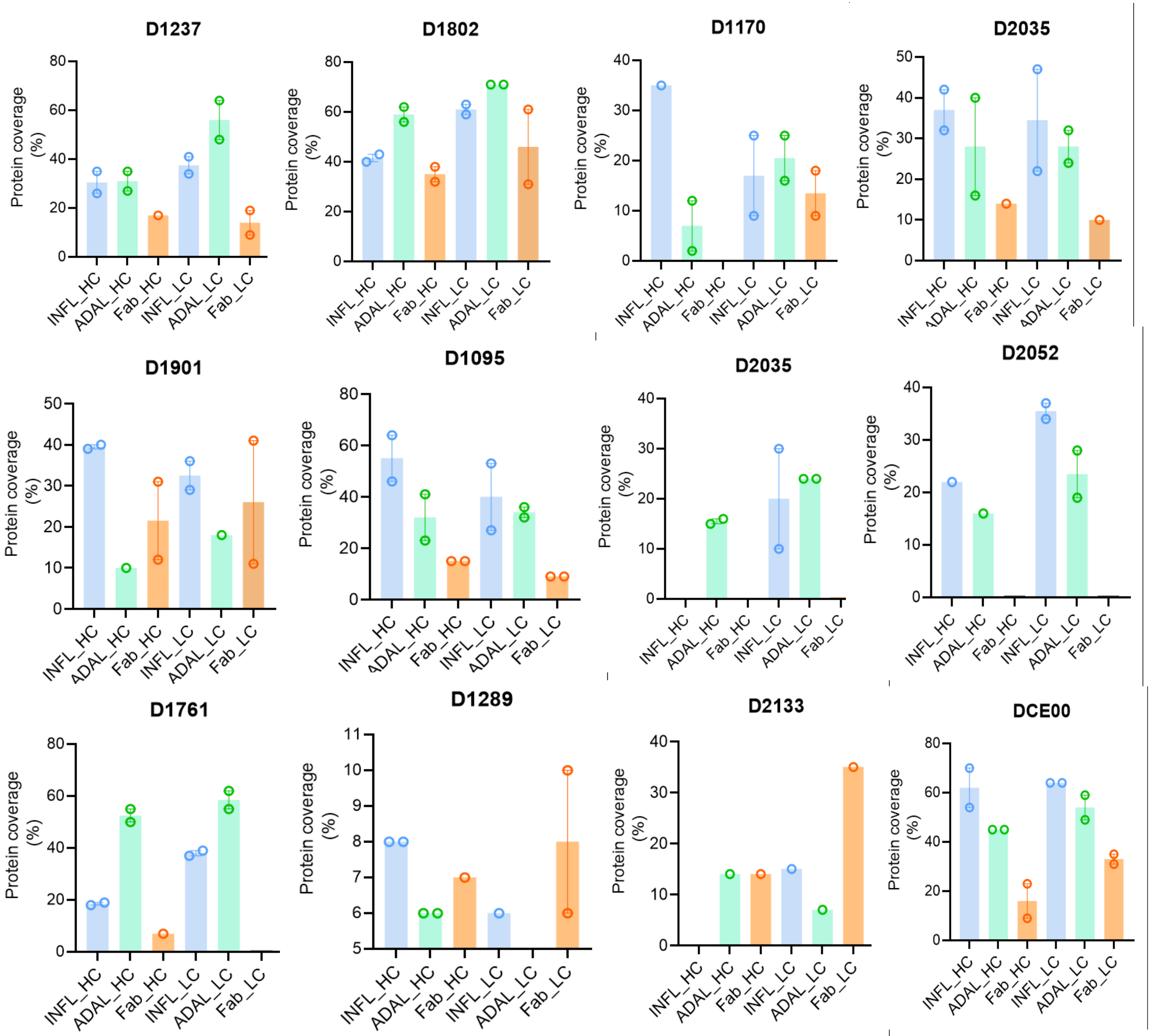

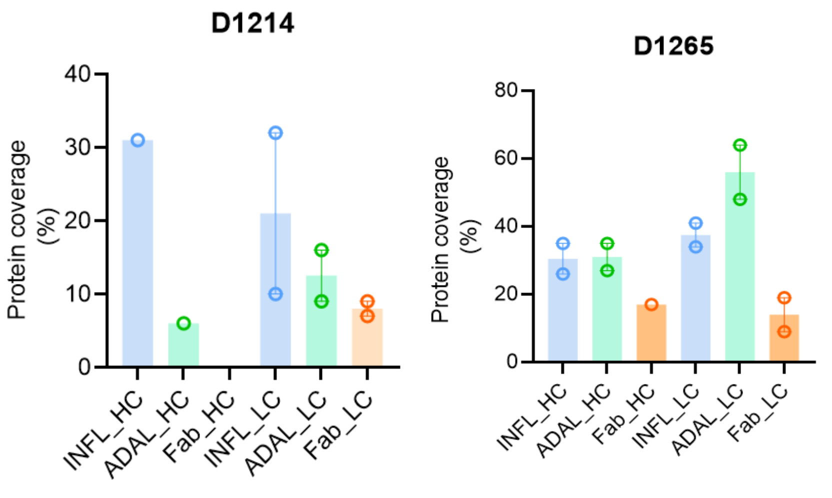

Supplementary Figure S7. Sequence coverage evaluation of HLAII-peptides from TNF antagonists identified in the 84 immunopeptidome-sets (14 donors; TNF antagonist and TNF immune complex conitions). Percentage of protein coverage spanning parental sequences from both light (LC) and heavy chains (HC) was plotted per antagonist and donor. Each bar represents the mean and SEM of two independent samples when detected.

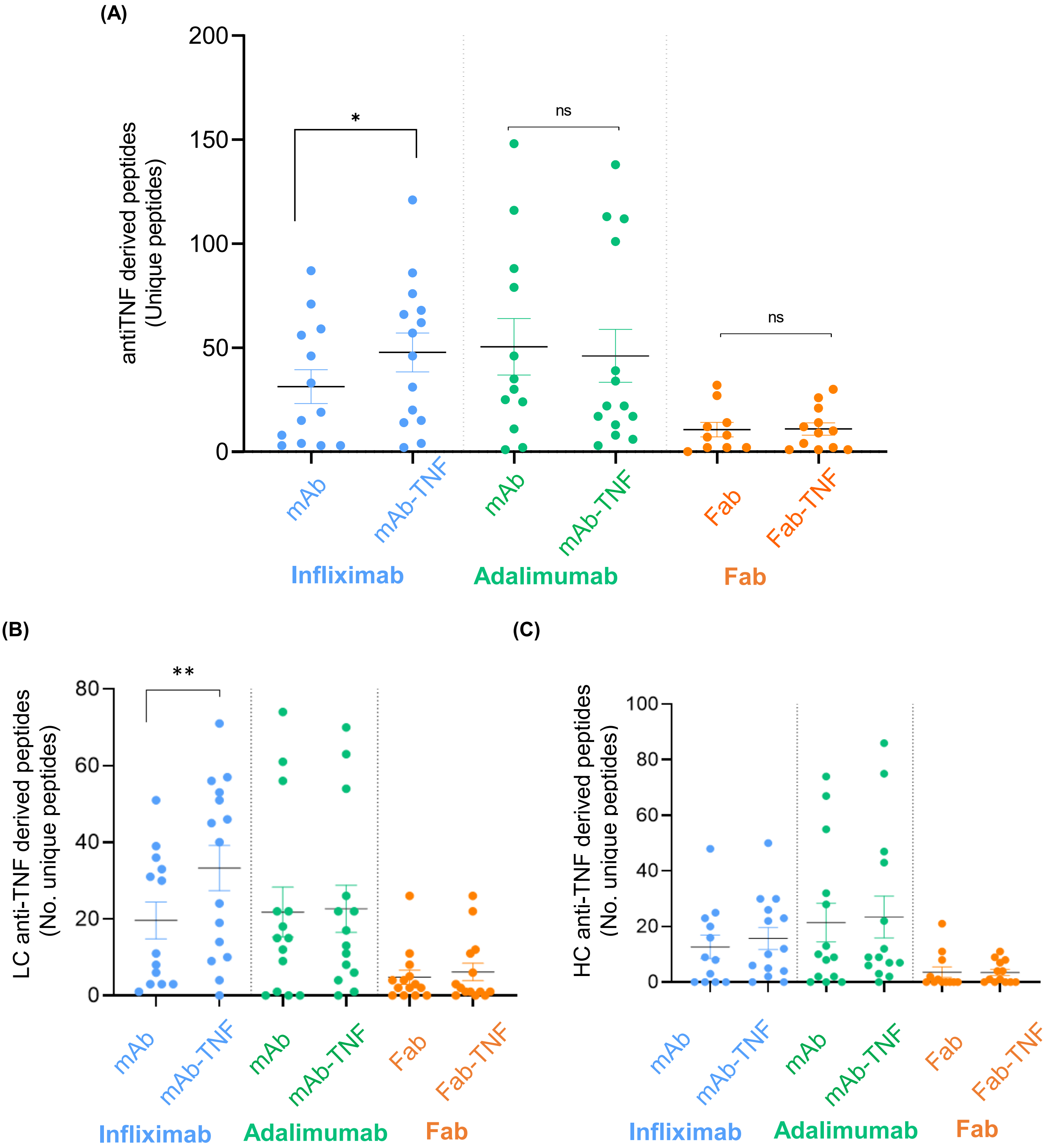

Supplementary Figure S8. HLAII-presented sequences derived from anti-TNF biotherapeutcis identified by HLAII immunopeptidomics (A) HLAII-peptides derived from all peptides from TNF-antagonists; i.e. light chain (LC) and heavy chain (HC) (*p<0.0134 by Wilcoxon marched-pairs signed rank test). Each set represents the mean (line) and the SEM of fourteen donor-derived immunopeptidomes (dots). **(B)** HLAII-peptides derived from the light chain (LC) of antagonists (**p<0.0085 by Wilcoxon marched-pairs signed rank test). **(C)** HLAII-peptides from the heavy chain (HC) of antagonists.

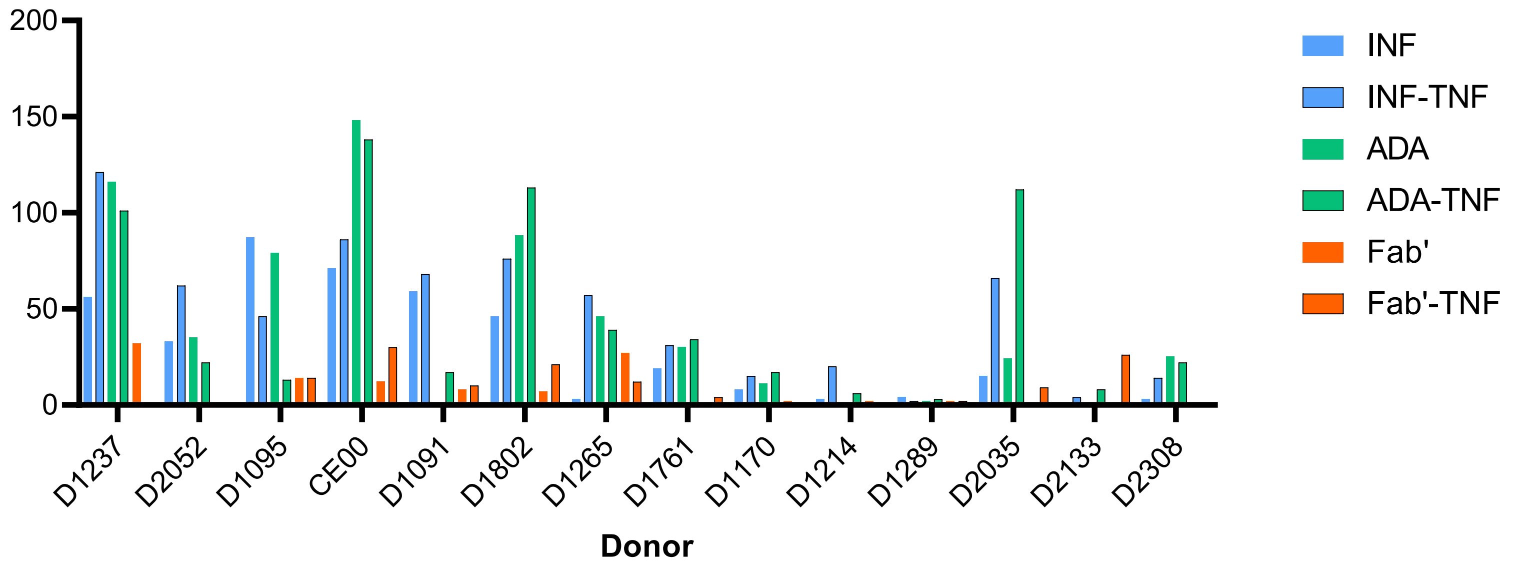

Supplementary Figure S9. Number of unique peptides derived from anti-TNF biotherapeutics per donor. Each bar represents No. of unique peptides from INFL (blue), ADAL (Green) and the single armed Fab' (orange),

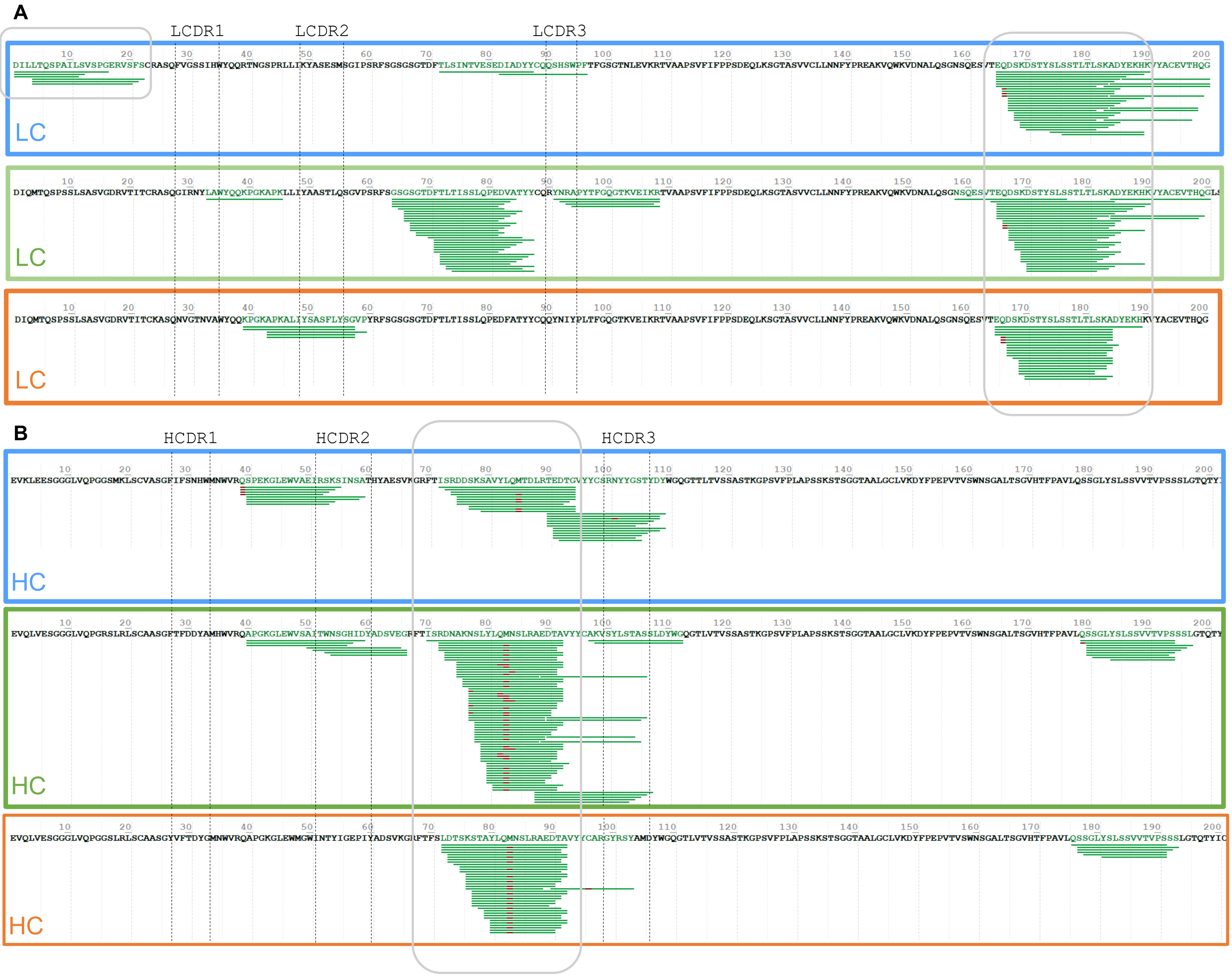

Supplementary Figure S10. Visualization of HLAII-presented peptides in INF (blue), ADAL (green) and Fab’ (orange) immunopeptidome sets from a representative donor (D1237). **A** LC-sequences aligned to identified ‘unique peptides’ (green lines) as mapped upon DB-search (protein coverage map, PMI-Byos). Grey boxes denote predominant nested sets detected in (≥8 donors). **B** HC-sequences aligned to identified ‘unique peptides’ (green lines). Hypervariable HCDR’s are presented (dashed lines).

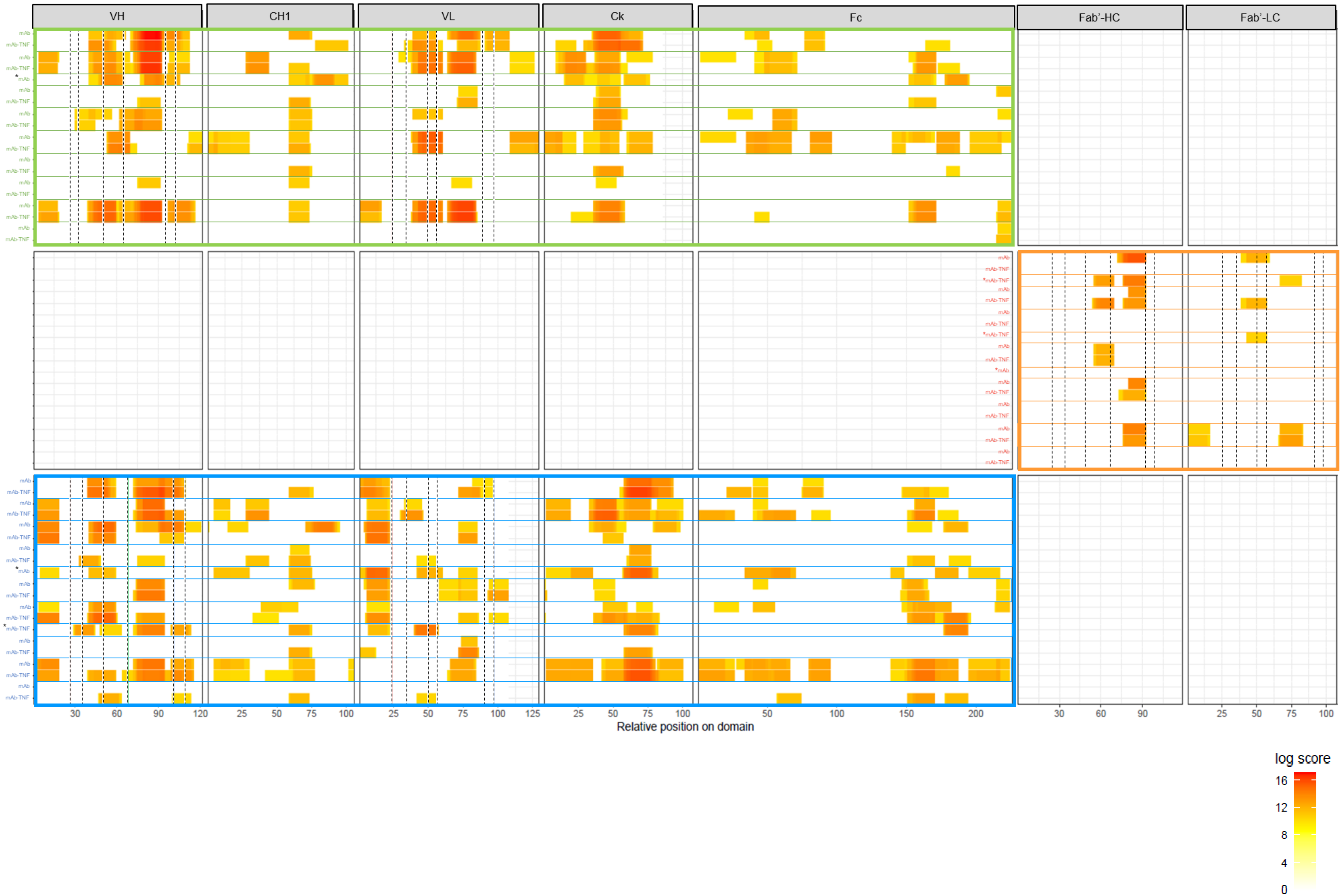
VH
CH1
VL
Ck
Fc
Fab'-HC
Fab'-LC
mAb
mAb-TNF
Relative position on domain
log score
16
12
8
4
0

Supplementary Figure S11. HeatMAPPS upon normalization of HLAII-immunopeptidomes by DataMAPPs (40). Visualization of HLAII-peptide-clusters detected by timsTOF in response to INF (blue), ADAL (green) and Fab' (orange) as well as TNF immune complexes, where a group of 2-, 3- and 2-sets from INFL, ADAL- and Fab', respectively, was excluded based on number of total peptides detected and Pearson's correlation results upon normalization as established quality criteria (40). Donor sets are presented in the following order: D1237, D1802, D1095, D1170, D1265, ,1761, D2035, D1901, D2052, CE00 and D1214, where * indicates those sets where one sample was excluded according the dataMAPPs established quality criteria. Dashed lines denote CDRs in VH and VL. Constant regions are also presented as CH, Ck and Fc for the bivalent antibodies ADA and ADAL.

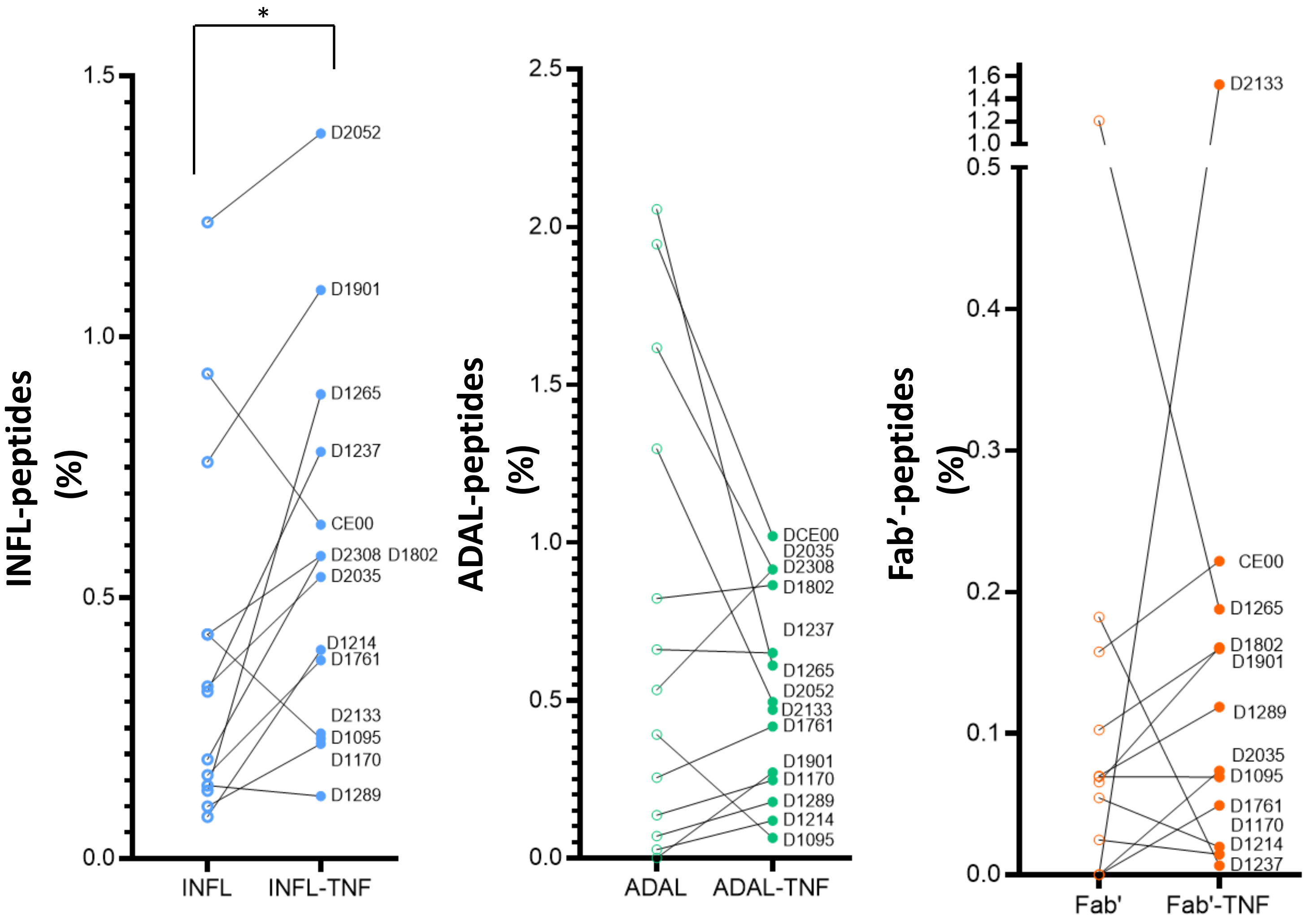

Supplementary Figure 12. HLAII-presented sequences derived from anti-TNF compounds identified by HLAII immunopeptidomics. Percentage of HLAII-peptides derived from test articles normalized to the total number of unique HLAII-peptides per sample, corresponding to INF (blue) ADAL (green) and Fab' (orange); *p<0.026 by Wilcoxon marched-pairs signed rank test.

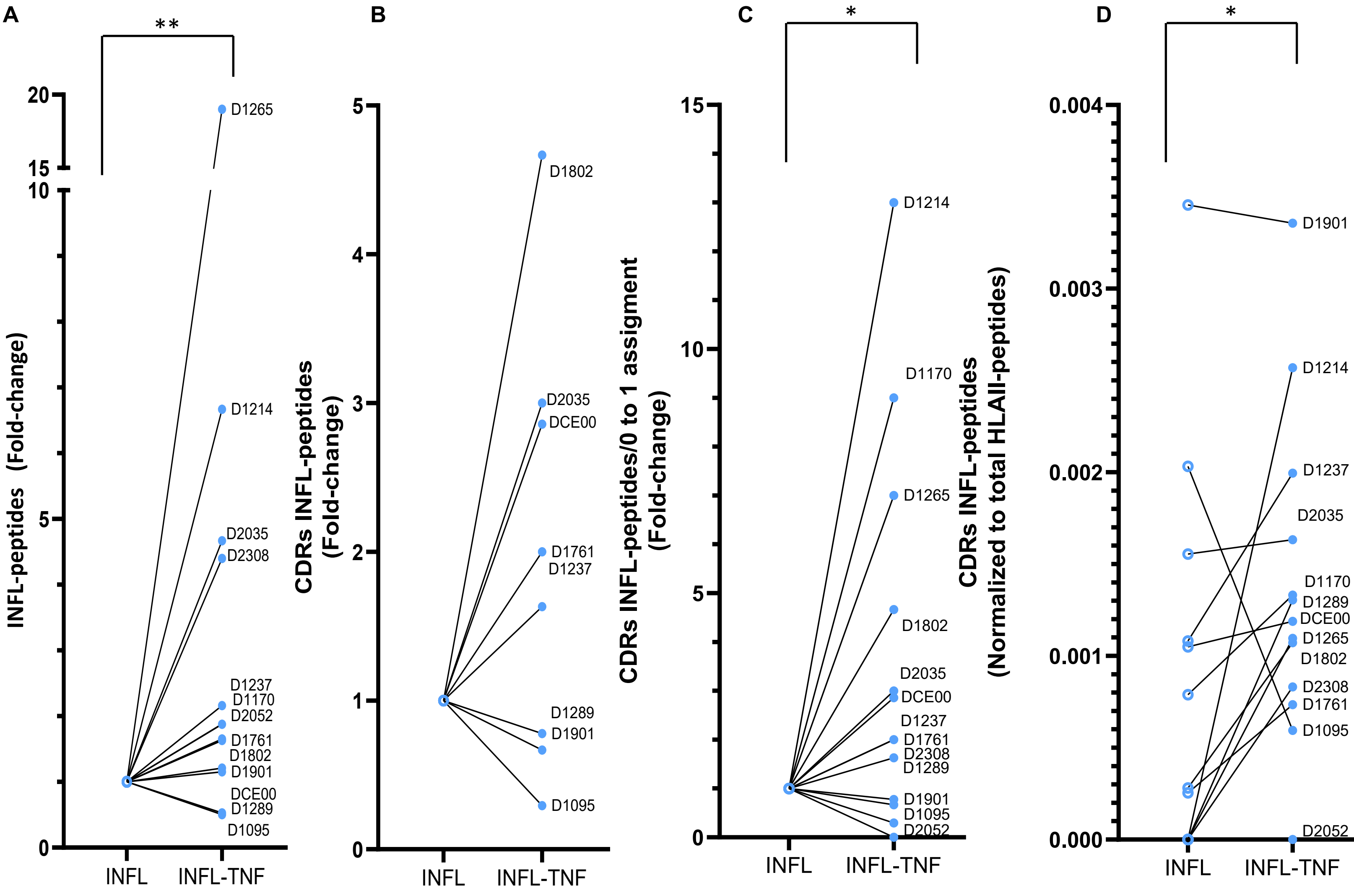

Supplementary Figure 13. HLAII-presented sequences derived from INF compounds identified by HLAII immunopeptidomics. Fold-increase of total INFL-peptides (A) and peptides including CDR-residue (B) in INFL-TNF relative to INFL samples (**p<0.0046 by Wilcoxon marched-pairs signed rank test). (C) Fold-increase of CDRs INFL-sequences with '1' peptide assignment for donors with undetected peptides in INFL sample (D1214, D1170, D1265; (*p<0.0215 by Wilcoxon marched-pairs signed rank test). (D) Normalized INFL-peptides to the total of unique peptides detected per sample (*p<0.046 by Wilcoxon marched-pairs signed rank test).
